# Upregulation of acid ceramidase contributes to tumor progression in tuberous sclerosis complex

**DOI:** 10.1172/jci.insight.166850

**Published:** 2023-05-08

**Authors:** Aristotelis Astrinidis, Chenggang Li, Erik Y. Zhang, Xueheng Zhao, Shuyang Zhao, Minzhe Guo, Tasnim Olatoke, Ushodaya Mattam, Rong Huang, Alan G. Zhang, Lori Pitstick, Elizabeth J. Kopras, Nishant Gupta, Roman Jandarov, Eric P. Smith, Elizabeth Fugate, Diana Lindquist, Maciej M. Markiewski, Magdalena Karbowniczek, Kathryn A. Wikenheiser-Brokamp, Kenneth D. R. Setchell, Francis X. McCormack, Yan Xu, Jane J. Yu

**Affiliations:** 1 Department of Internal Medicine, University of Cincinnati College of Medicine, Cincinnati, Ohio, USA.; 2 Clinical Mass Spectrometry Laboratory, Division of Pathology and Laboratory Medicine, Cincinnati Children’s Hospital Medical Center, Cincinnati, Ohio, USA.; 3 Department of Pediatrics, University of Cincinnati College of Medicine, Cincinnati, Ohio, USA.; 4 Divisions of Pulmonary Biology and Biomedical Informatics, Perinatal Institute, Cincinnati Children’s Hospital Medical Center, Cincinnati, Ohio, USA.; 5 Department of Environmental and Public Health Sciences, University of Cincinnati College of Medicine, Cincinnati, Ohio, USA.; 6 Department of Radiology, Cincinnati Children’s Hospital Medical Center, Cincinnati, Ohio, USA.; 7 Department of Immunotherapeutics and Biotechnology, Jerry H. Hodge School of Pharmacy, Texas Tech University Health Sciences Center, Abilene, Texas, USA.; 8 Division of Pathology and Laboratory Medicine; Division of Pulmonary Medicine; and Division of Pulmonary Biology, Section of Neonatology, Perinatal and Pulmonary Biology, Perinatal Institute, Cincinnati Children’s Hospital Medical Center, Cincinnati, Ohio, USA.; 9 Department of Pathology and Laboratory Medicine, University of Cincinnati College of Medicine, Cincinnati, Ohio, USA.

**Keywords:** Cell Biology, Metabolism, Molecular biology, Mouse models, Tumor suppressors

## Abstract

Tuberous sclerosis complex (TSC) is characterized by multisystem, low-grade neoplasia involving the lung, kidneys, brain, and heart. Lymphangioleiomyomatosis (LAM) is a progressive pulmonary disease affecting almost exclusively women. TSC and LAM are both caused by mutations in *TSC1* and *TSC2* that result in mTORC1 hyperactivation. Here, we report that single-cell RNA sequencing of LAM lungs identified activation of genes in the sphingolipid biosynthesis pathway. Accordingly, the expression of acid ceramidase (*ASAH1*) and dihydroceramide desaturase (*DEGS1*), key enzymes controlling sphingolipid and ceramide metabolism, was significantly increased in TSC2-null cells. TSC2 negatively regulated the biosynthesis of tumorigenic sphingolipids, and suppression of ASAH1 by shRNA or the inhibitor ARN14976 (17a) resulted in markedly decreased TSC2-null cell viability. In vivo, 17a significantly decreased the growth of TSC2-null cell–derived mouse xenografts and short-term lung colonization by TSC2-null cells. Combined rapamycin and 17a treatment synergistically inhibited renal cystadenoma growth in *Tsc2*^+/–^ mice, consistent with increased ASAH1 expression and activity being rapamycin insensitive. Collectively, the present study identifies rapamycin-insensitive ASAH1 upregulation in TSC2-null cells and tumors and provides evidence that targeting aberrant sphingolipid biosynthesis pathways has potential therapeutic value in mechanistic target of rapamycin complex 1–hyperactive neoplasms, including TSC and LAM.

## Graphical abstract

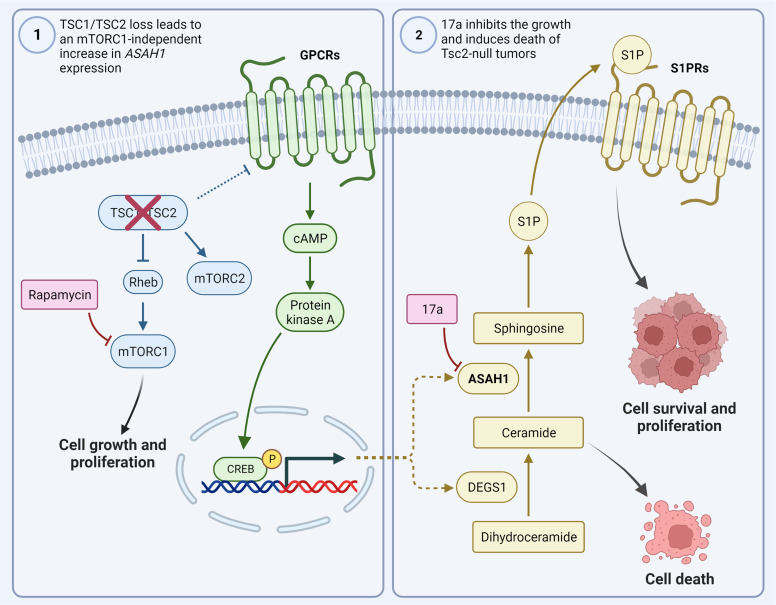

## Introduction

Tuberous sclerosis complex (TSC) is an autosomal dominant disorder with multisystem manifestations, including low-grade neoplasms in the brain, heart, lungs, and kidneys (1–3). Complications associated with TSC tumors include cognitive impairment, intractable seizures, autism, and renal hemorrhage and insufficiency. Lymphangioleiomyomatosis (LAM) is another TSC deficiency disorder resulting in progressive lung disease affecting almost exclusively women (4). LAM presents with fatigue, dyspnea, pneumothorax, and progressive loss of pulmonary function. Approximately 50% of women with TSC and 10% of men with TSC have cystic changes on chest radiology consistent with LAM (5–8). TSC and LAM are caused by inactivating mutations in *TSC1* or *TSC2* (9–11), which encode hamartin and tuberin, respectively. Hamartin and tuberin form a complex with GTPase, activating protein activity toward Ras homolog enriched in brain (Rheb), an activator of the mechanistic target of rapamycin complex 1 (mTORC1) (12–14). Thus, loss-of-function mutations in either *TSC1* or *TSC2* result in constitutive activation of mTORC1. mTOR is a serine/threonine protein kinase that functions as the catalytic subunit in 2 complexes, mTORC1 and mTORC2 (15). mTORC1 contains Raptor and phosphorylates several substrates, including ribosomal protein S6 kinase, translational repressor 4E-binding protein 1 (4E-BP1), and Unc-51-like kinase 1 (ULK1), thereby regulating ribosome biogenesis, mRNA translation, protein synthesis, autophagy, cell metabolism, gene transcription, and cell growth (16–18). mTORC2 contains Rictor and phosphorylates protein kinases, including PKC and AKT at Ser473, to control cell survival, cytoskeletal organization, and cell motility (19).

The discovery that *TSC1* or *TSC2* mutations cause dysregulation of mTORC1/mTORC2 led to preclinical studies (20–25) and human clinical trials demonstrating therapeutic efficacy of mTORC1 inhibitors (mTORi) in TSC and LAM (26–31). However, the use of mTORi in LAM and TSC has several limitations, including 1) the effect on tumor growth is cytostatic rather than cytotoxic; 2) tumors typically regrow upon treatment cessation (26, 28, 32–34); 3) some mTOR-related disease manifestations are poorly responsive to therapy; and 4) patients are at significant risk for potential side effects given the need for long-term treatment. Thus, there is a need to optimize mTORi approaches and to develop novel remission-inducing therapies.

We previously reported that prostaglandin biosynthesis and functions are dysregulated in TSC2-deficient cells (35–38). Prostaglandin E_2_ receptors (EP1–4) are G protein–coupled receptors that integrate signals to regulate various cellular functions (39, 40), including cAMP production and transactivation of gene expression, including *N*-acylsphingosine amidohydrolase 1 (acid ceramidase, *ASAH1*) (41). We found that TSC2-null angiomyolipoma-derived (AML-derived) cells produce excessive prostaglandin E_2_ (PGE_2_) induced by COX2 and express higher levels of PGE_2_ receptor 3 (EP3) relative to TSC2-reexpressing cells (35–38). These results suggest an interplay between PGE_2_ activity and sphingolipid metabolism in TSC-deficient tumors. Sphingolipids are major components of the eukaryotic plasma membrane that mediate multiple essential cellular functions, including apoptosis, proliferation, stress responses, necrosis, inflammation, autophagy, senescence, tumor growth (42, 43), cancer progression, and differentiation (43). Ceramide and sphingosine-1-phosphate, the 2 central bioactive sphingolipids, exhibit opposing roles in regulating cancer cell death and survival (43–45). Ceramide is considered to be at the core of sphingolipid metabolism; intracellular concentrations of this intermediary directly increase apoptosis and cell cycle arrest (46). The rate-limiting step of de novo ceramide synthesis is the condensation of palmitate and serine by serine palmitoyl transferase. The final reaction to produce ceramide from dihydroceramide is catalyzed by delta 4-desaturase sphingolipid 1 (dihydroceramide desaturase, DEGS1). ASAH1 catalyzes the cleavage of ceramide to sphingosine and fatty acids and controls the sphingosine/ceramide ratio (43, 46). Carmofur, a derivative of 5-fluorouracil, is a broad-spectrum antimetabolite that affects a wide range of pathways. Studies of carmofur activity in colorectal cancer cells (47), patients with colorectal carcinoma (48), glioblastoma cells (49), and breast cancer cells (50) demonstrate that carmofur is effective in suppressing ASAH1 expression.

Here, we report the identification of sphingolipid biosynthesis pathway genes in disease-defining LAM lung cells by single-cell RNA sequencing. Analysis of publicly available expression array data from TSC2-null AML-derived cells (51) revealed that *DEGS1* and *ASAH1* transcripts were significantly increased in TSC2-null AML-derived cells, compared with TSC2-reexpressing cells. Moreover, we discovered TSC2-dependent upregulation of *DEGS1* and *ASAH1* expression in cell models of human AML-derived cells, clinical renal AMLs, and pulmonary LAM patient specimens. Mechanistic studies showed that enhanced expression of DEGS1 and ASAH1 was independent of mTORC1 inhibition, which is potentially novel and previously unexplored. Together, our studies identify rapamycin-insensitive upregulation of enzymes contributing to sphingolipid metabolism in TSC2-null cells and tumors. Our studies reveal a potentially novel function of TSC2 that negatively regulates sphingolipid production and action via ASAH1. Thus, targeting aberrant sphingolipid biosynthesis pathways has potential therapeutic value in patients with TSC and LAM, as well as possibly in other mTORC1-hyperactive neoplasms.

## Results

### Single-cell RNA sequencing identifies sphingolipid biosynthesis pathway gene activation in disease-defining LAM lung lesion cells.

**Figure 1:**
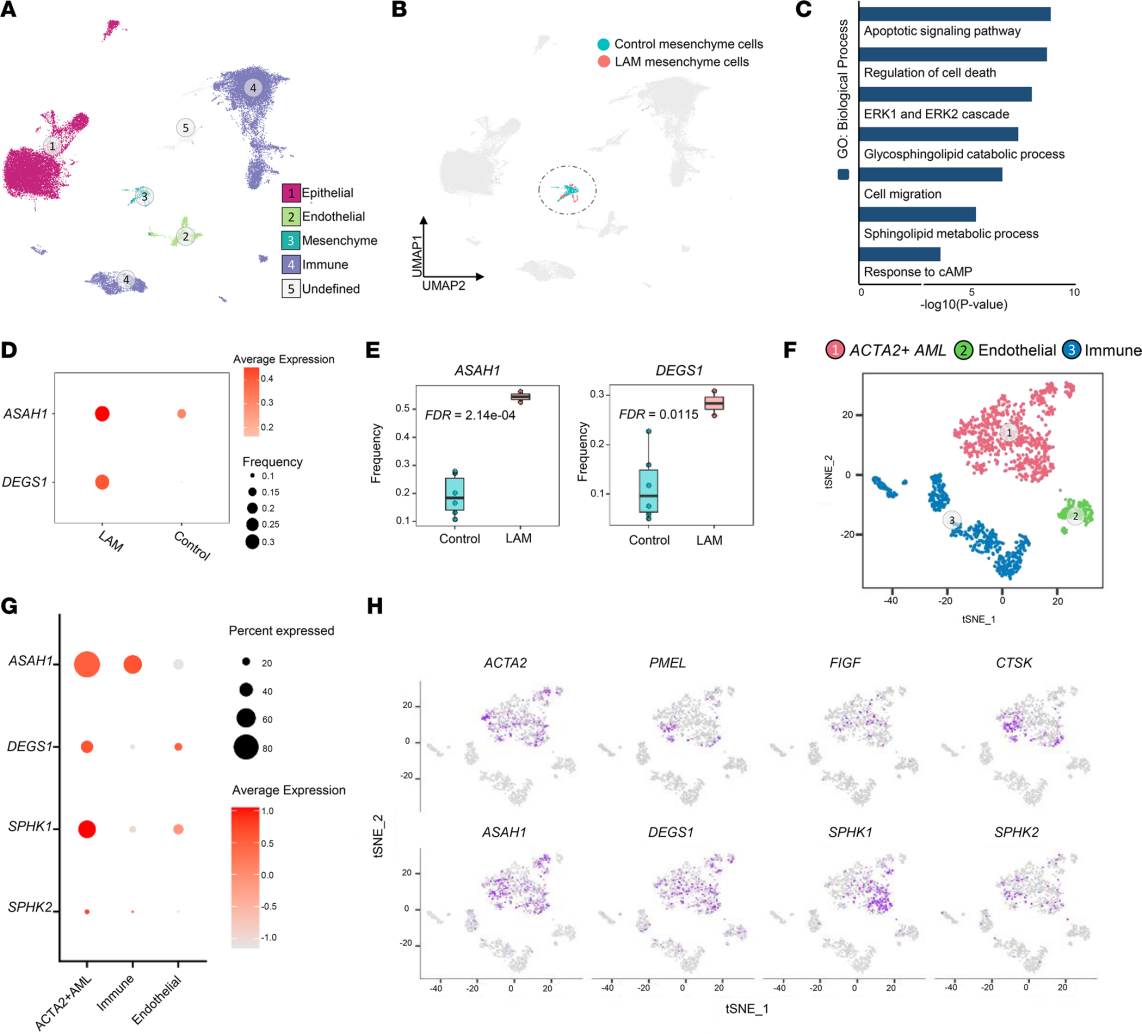
scRNA-Seq analysis identified sphingolipid pathways and related genes induced in LAM mesenchymal cells and renal AML ACTA2^+^ cells. (**A**) Integration of 54,511 cells from 2 LAM lung samples and 6 control lung samples. Cells are visualized using uniform manifold approximation and projection and colored by major lineages. (**B**) A total of 736 mesenchymal cells from control samples and 190 mesenchymal cells from LAM lung samples are integrated and extracted for direct comparison. (**C**) Functional enrichment analysis of genes induced in LAM mesenchymal cells versus control mesenchymal cells. (**D**) Dot plots showing the increasing expression and frequency of ASAH1 and DEGS1 in LAM versus control. Node size represents gene expression frequency. Node color represents the scaled average expression. (**E**) Box plots showing the expression frequency of representative sphingolipid biosynthesis pathway genes in control mesenchymal cells and LAM mesenchymal cells. Box plots show the interquartile range (box), median (line), and minimum and maximum (whiskers). (**F**) Visualization of 1,583 cells from lesions of renal AML tumor cells. Cells are visualized using t-distributed stochastic neighbor embedding. Cells are colored by condition. AML lesions consist of 3 major cell types: ACTA2^+^ AML cells (largest cluster), immune cells, and endothelial cells. AML scRNA-Seq data were downloaded from GEO GSM4035469, with cell type annotation based on the previous study (52). (**G**) Dot plot showing selected sphingolipid biosynthesis pathway genes’ expression comparison across the 3 AML cell populations. Node size represents gene expression frequency. Node color represents the scaled average expression. (**H**) Feature plots of LAM and AML markers (*ACTA2*, *PMEL*, *FIGF*, and *CTSK*) and sphingolipid pathway genes (*ASAH1*, *DEGS1*, *SPHK1*, and *SPHK2*) in AML cells.

We previously employed single-cell RNA sequencing (scRNA-Seq) and systems biology tools to identify LAM cells, define their signature genes and enriched functions, and delineate altered signaling pathways associated with LAM pathogenesis (52). In the present study, we integrated scRNA-Seq of LAM lungs (*n* = 2, National Center for Biotechnology Information Gene Expression Omnibus [GEO] GSE135851) and control female lung samples (*n* = 6, GSE122960), resulting in a total of 54,511 cells that were clustered into distinct cell lineages based on expression of lineage-specific markers (epithelium: EPCAM and CDH1; endothelium: CDH5 and EMCN; mesenchymal: TCF21 and actin alpha 2, smooth muscle [ACTA2]; immune: PTPRC and CD68) (Figure 1A). Differentially expressed genes were identified by comparing 190 mesenchymal cells from LAM lung samples with 736 mesenchymal cells from control samples (Figure 1B) and subjected to gene set enrichment analysis. Gene set enrichment analysis of genes included in LAM lung cells revealed significant enrichment of sphingolipid metabolic process, glycosphingolipid catabolic process, and response to cAMP in LAM versus control (Figure 1C). Among these genes, *ASAH1* and *DEGS1* mRNA expression levels and frequency of *ASAH1*- and *DEGS1*-positive cells within the LAM mesenchymal clusters were significantly increased relative to control mesenchymal cells (Figure 1, D and E). In addition, we analyzed scRNA-Seq data from one renal AML harboring a *TSC1* mutation (52). Visualization of 1,583 cells from the AML lesion showed 3 major cell types: ACTA2^+^ AML cells (cluster 1), endothelial cells (cluster 2, and immune cells (cluster 3) (Figure 1F). A dot plot revealed abundant expression of selected sphingolipid pathway genes *ASAH1*, *DEGS1*, *SPHK1*, and *SPHK2* in ACTA2^+^ AML cells (Figure 1G). Feature plots of ACTA2^+^ AML cells showed selective expression of known LAM and AML markers, including *ACTA2*, *PMEL*, *FIGF*, and *CTSK*, and several key sphingolipid pathway genes, including *ASAH1*, *DEGS1*, *SPHK1*, and *SPHK2*, were selectively expressed in the same ACTA2*^+^* AML cell cluster (Figure 1H). Collectively, our scRNA-Seq analysis identified selective activation of *ASAH1* and *DEGS1* genes in LAM lung and renal AML cells worthy of further investigation.

### Differential expression of acid ceramidase ASAH1 is evident in LAM lesions.

**Figure 2:**
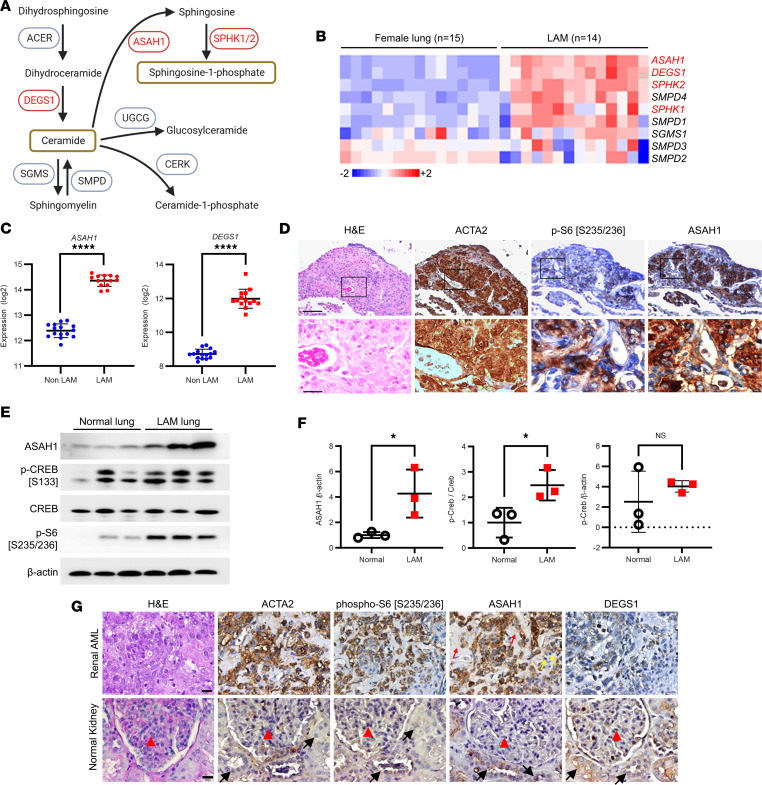
Expression of DEGS1 and ASAH1 is evident in pulmonary LAM lesions. (**A**) Sphingolipid biosynthesis pathway shows genes with upregulated expression in TSC2-null cells (in red). (**B**) Heatmap of sphingolipid metabolism genes expression in female non-LAM lungs (*n* = 15 subjects) and laser capture microdissected LAM lesion cells (*n* = 14 subjects). (**C**) *ASAH1* expression in LAM cells, compared with control female non-LAM lungs. ****P < 0.0001, Mann-Whitney test. (**D**) Immunohistochemistry of hematoxylin and eosin (H&E), smooth muscle actin (ACTA2), phosphorylated S6 (p-S6, Ser235/236), and ASAH1 in LAM lung tissues. Representative images of 3 cases are shown. Scale bars are 100 μm and 20 μm for the top and bottom rows, respectively. (**E**) Immunoblotting of ASAH1 and transcription factor cAMP-responsive element-binding protein (CREB) in LAM and control lungs (*n* = 3). (**F**) Densitometry of ASAH1 and p-CREB (*n* = 3). *P < 0.05, unpaired *t* test. (**G**) H&E, ACTA2, p-S6 (Ser235/236), ASAH1, and DEGS1 in renal AML and normal kidney. Representative images of 3 cases are shown. Scale bars are 20 μm. ACTA2-positive renal AML cells (brown staining), stroma (thin red arrows), infiltrating mononuclear cells (thin yellow arrows), normal glomeruli (red arrowheads), and tubular epithelial cells (black arrows).

Activation of the sphingolipid biosynthesis pathway leads to sphingosine production and bioactive lipid signaling molecule activation, which positively affects cell viability and tumor progression (43, 46). Note that AMLs have “mesenchymal” characteristics, containing immature smooth muscle cells, adipose cells, and aberrant blood vessels (53). To determine whether the sphingolipid biosynthesis pathway (Figure 2A) is activated in LAM, we compared gene expression data from non-LAM female lungs (*n* = 15) with LAM lungs (*n* = 14) and found that *ASAH1* and *DEGS1* transcript levels were significantly higher in LAM (Figure 2, B and C; Supplemental Figure 1; and Supplemental Table 1; supplemental material available online with this article; https://doi.org/10.1172/jci.insight.166850DS1; *P* < 0.0001 Mann-Whitney test). To determine the protein levels of ASAH1 in human lung lesions, we performed immunohistochemical staining in LAM lung tissues obtained from the National Disease Research Exchange. ASAH1 protein colocalized with smooth muscle actin–positive and phospho-S6–positive cells within LAM lung lesions, with ASAH1 being upregulated in LAM lesions, compared with adjacent normal tissue (Figure 2D). Immunoblot analysis showed a 4-fold increase in ASAH1/β-actin in the LAM lung tissue lysates, compared with normal lung (Figure 2, E and F; Supplemental Figure 2; and Supplemental Tables 1 and 2; unpaired *t* test *P* < 0.05). Since ASAH1 expression is regulated by CREB (54), we tested CREB activation by immunoblotting with a p-CREB (Ser133) antibody, demonstrating a 2.5-fold increase in p-CREB/CREB ratio (Figure 2, E and F; Supplemental Figure 2; and Supplemental Tables 1 and 2; unpaired *t* test *P* < 0.05). Finally, we assessed DEGS1 and ASAH1 proteins in renal AML and normal kidney. ASAH1 expression was evident in ACTA2^+^ renal AML cells (brown staining). Stroma and infiltrating mononuclear cells were negative (Figure 2G). We also found that both ASAH1 and DEGS1 colocalized with ACTA2-positive and p-S6–positive cells (Figure 2G). DEGS1 accumulation was present in a subpopulation of ACTA2^+^ renal AML cells. Normal glomeruli and tubular epithelial cells in the normal kidney lacked specific expression of ACTA2 and p-S6. Minimal DEGS1 staining was present in glomerular and tubular epithelial cells (Figure 2G). Normal glomeruli contain an abundance of mesenchymal origin mesangial cells that provide support for the glomerulus capillary network (55). Therefore, our controls contained epithelial and mesenchymal components of normal kidney, and expression of ASAH1 and DEGS1 was evaluated in both components. ASAH1 staining was negative in glomeruli, whereas some epithelial tubular cells expressed low levels of ASAH1. DEGS1 seemed to be expressed in some glomerular and tubular cells (Figure 2G). Collectively, these data provide evidence that the sphingolipid biosynthesis pathway proteins are evident in TSC2-null cells as well as LAM lung and kidney AML lesions.

### TSC2 negatively regulates ASAH1 expression.

**Figure 3:**
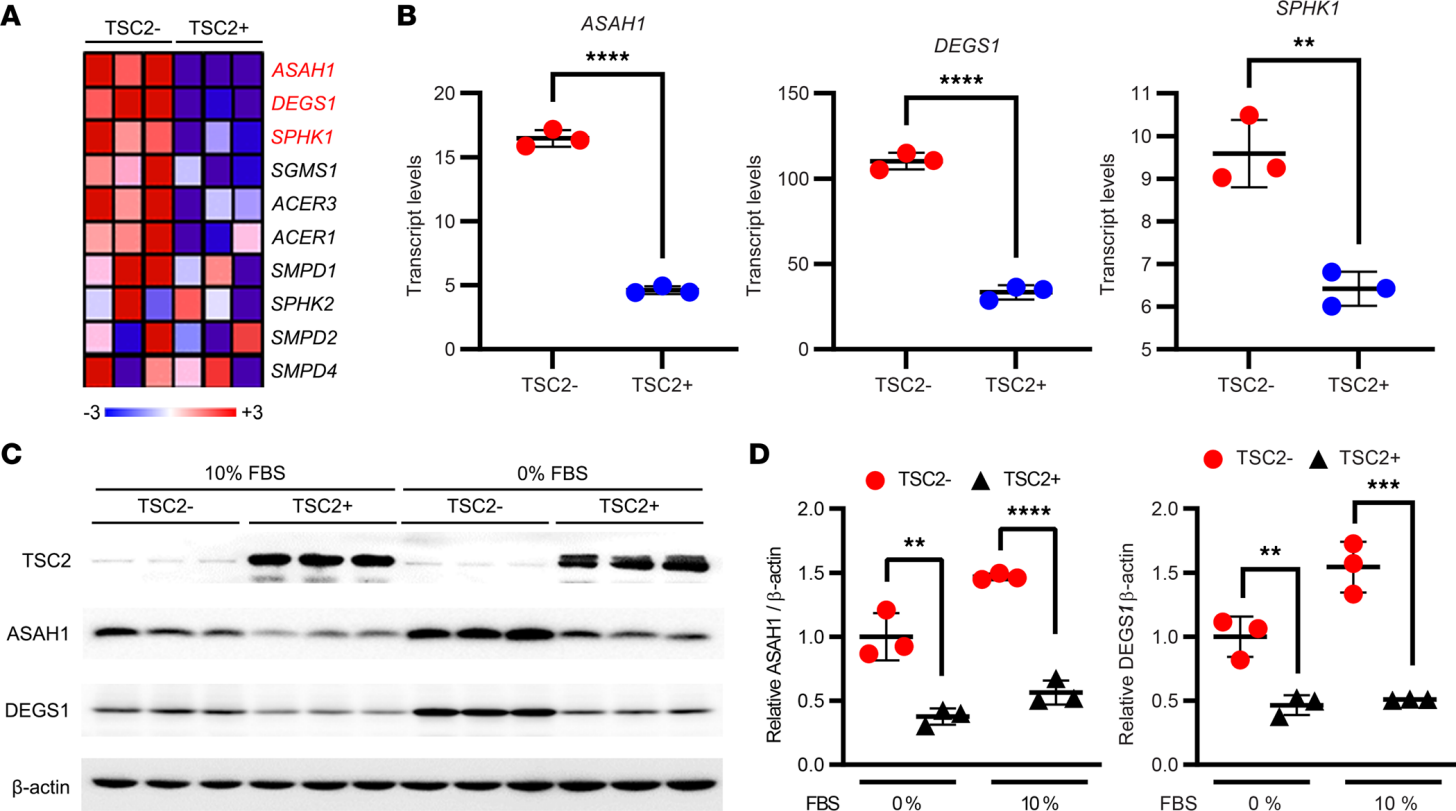
Upregulation of ASAH1 and DEGS1 expression in TSC2-null LAM-derived cells. (**A**) Heatmap of the expression of sphingolipid biosynthesis pathway genes in TSC2-null (TSC2-) 621-102 and TSC2-addback (TSC2+) 621-103 cells. The scale indicates the fold-change of genes from blue (min) to red (max) (–3 to +3). (**B**) Transcript levels of *ASAH1*, *DEGS1*, and *SPHK1* in TSC2-null patient-derived 621-102 and 621-103 cells. (**C**) The protein levels of TSC2, DEGS1, and ASAH1 were assessed by immunoblotting. β-Actin was used as a loading control. (**D**) Densitometry of ASAH1 and DEGS1 protein levels normalized to β-actin (*n* = 3/group). (**B** and **D**) **P < 0.01, ***P < 0.001, ****P < 0.0001, unpaired *t* test.

To determine whether sphingolipid biosynthesis pathway gene expression is regulated by TSC2, we reanalyzed previously published genomic data from TSC2-null AML-derived cells — 621-102 (51) — and found that genes participating in the sphingolipid pathway were upregulated in TSC2-null cells (Figure 3A), including *ASAH1*, *DEGS1*, and *SPHK1* transcripts, compared with TSC2-addback 621-103 cells (Figure 3B, Supplemental Figure 3, and Supplemental Tables 1 and 3; *ASAH1* *P* < 0.0001, *DEGS1* *P* < 0.0001, and *SPHK1* *P* = 0.0034 by unpaired *t* test). Immunoblotting analysis showed that DEGS1 and ASAH1 proteins were also more abundant in TSC2-null 621-101 relative to TSC2-addback 621-103 cells, in both regular (10% FBS) and serum-free (0% FBS) culture conditions (Figure 3, C and D; Supplemental Figure 3; and Supplemental Tables 1 and 4). These findings indicate aberrant upregulation of the sphingolipid biosynthesis pathway in TSC2-null AML-derived cells.

### Cellular levels of sphingosine are elevated in a rapamycin-insensitive manner in LAM-derived cells.

**Figure 4:**
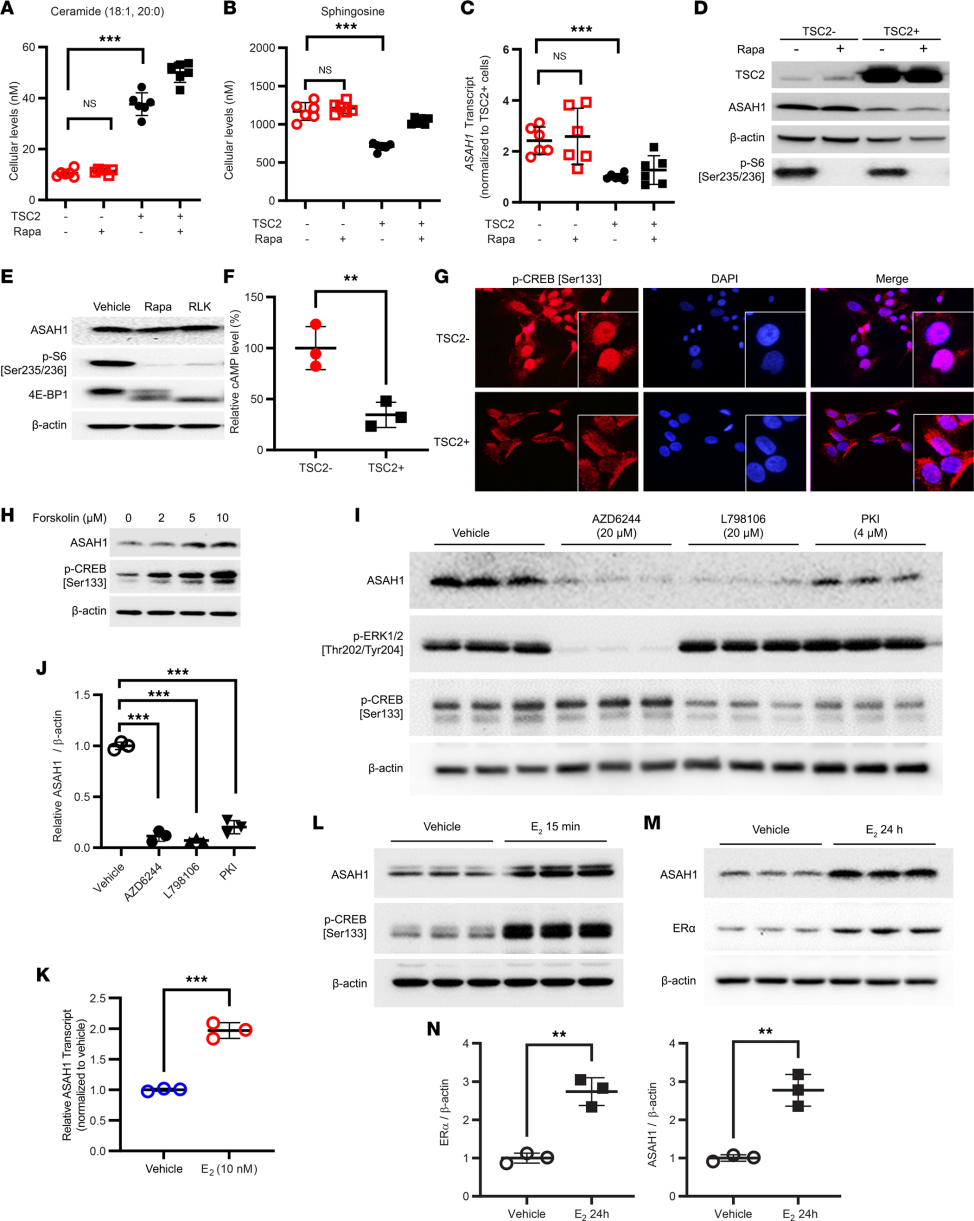
ASAH1 activity and expression are elevated in a sirolimus-insensitive manner in LAM-derived cells. 621-101 (TSC2-) and 621-103 (TSC2+) cells were treated with 20 nM sirolimus (Rapa) for 24 hours. Cellular levels of ceramide (**A**) and sphingosine (**B**) were quantified using LC-MS/MS. (**C**) *ASAH1* transcript levels were quantified by qRT-PCR. (**D** and **E**) LAM patient–derived 621-101 (TSC2-) and 621-103 (TSC2+) cells were treated with mTORC1 inhibitor rapamycin (Rapa) (20 nM) or rapalink-1 (RLK1) (0.1 μM) for 24 hours. Protein levels of TSC2, ASAH1, p-S6 (Ser235/236), and 4E-BP1 were assessed by immunoblotting. β-Actin was used as a loading control. (**F**) Cellular levels of cAMP were quantified in 621-101 (TSC2-) and 621-103 (TSC2+) cells (*n* = 3). (**G**) Representative images of confocal microscopy of p-CREB are shown. Nuclei were stained with DAPI. Orginial magnification, ×200. Inset magnification, ×400. (**H** and **I**) 621-101 (TSC2-) cells were treated with cAMP agonist forskolin (2, 5, and 10 μM), MEK1/2 inhibitor AZD6244 (20 μM), EP3 inhibitor L798106 (20 μM), or protein kinase A inhibitor (PKI) (4 μM), for 24 hours. Protein levels of ASAH1, p-CREB (S133), and p-Erk1/2 were assessed by immunoblotting. β-Actin was used as a loading control. (**J**) Densitometry of ASAH1 protein levels normalized to β-actin (*n* = 3). (**K**–**M**) 621-101 (TSC2-) cells were treated with 10 nM E2 for 15 minutes or 24 hours. *ASAH1* transcript levels were quantified using qRT-PCR. Immunoblot analyses of p-CREB (Ser133), ASAH1, and estrogen receptor-α (ERα) were performed (*n* = 3/group). β-Actin was used as a loading control. (**N**) Densitometry of ERα and ASAH1 protein levels (*n* = 3). (**A**–**C**, **F**, **J**, **K**, and **N**) **P < 0.01, ***P < 0.001. (**A**–**C** and **J**) Unpaired *t* test with Bonferroni’s multiple-comparison adjustment. (**F**, **K**, and **N**) Unpaired *t* test.

ASAH1 catalyzes the cleavage of ceramide to sphingosine. To determine whether the elevated ASAH1 expression in TSC2-null cells correlates with its enzymatic activity, we measured cellular levels of ceramides and sphingosine using liquid chromatography-tandem mass spectrometry (LC-MS/MS). In TSC2-null cells the levels of ceramide were 3-fold lower (Figure 4A), and the levels of sphingosine were 2-fold higher (Figure 4B), compared with TSC2-addback cells. Importantly, rapamycin treatment did not alter the levels of ceramides or sphingosine in TSC2-null cells, suggesting a rapamycin-insensitive regulation of ceramide conversion to sphingosine. Moreover, quantitative Reserve Transcription (qRT-PCR) analysis showed that *ASAH1* mRNA levels were 2.4-fold higher in TSC2-null cells, compared with TSC2-addback cells, and rapamycin did not affect *ASAH1* mRNA levels (Figure 4C). Furthermore, ASAH1 protein levels were higher in TSC2-null cells, compared with TSC2-addback cells, and rapamycin did not alter ASAH1 protein levels while suppressing S6 phosphorylation as expected (Figure 4D). We next treated TSC2-null cells with rapalink-1 (RLK1, 5 nM), a third-generation mTORi that is a more potent mTORi than rapamycin and other first- and second-generation mTORi. Although RLK1 treatment drastically suppressed phosphorylation of S6 and 4E-BP1, ASAH1 protein levels were not affected (Figure 4E). Collectively, our data indicate an mTOR-independent regulation of ASAH1 expression and sphingolipid biosynthesis in TSC2-null cells.

### ASAH1 expression is dependent on CREB and ERK activity.

cAMP inhibits mTORC1 signaling in a TSC1/TSC2-independent manner (56). CREB is tightly regulated by intracellular cAMP levels. Since we observed an upregulation of CREB phosphorylation in LAM lung tissue lysates (Figure 2, E and F), we assessed the cAMP levels in TSC2-null 621-101 and TSC2-reexpressing 621-103 cells and found that expression of TSC2 decreased intracellular cAMP levels by 3- to 4-fold (Figure 4F). Consistent with these observations, TSC2-null cells had increased nuclear p-CREB (Ser133), compared with TSC2-reexpressing cells, which showed a diffuse cytoplasmic staining (Figure 4G). To test whether increased CREB activation correlates with ASAH1 expression, we treated TSC2-null cells with forskolin, an adenyl cyclase activator that increases intracellular cAMP. ASAH1 protein levels were increased in a dose-dependent manner (Figure 4H). As expected, CREB phosphorylation was also increased by forskolin. Since activation of various pathways, including MAPK, PKA, prostaglandins, and estrogen (57), increases phosphorylation of CREB, we tested whether perturbations of these pathways lead to alterations in ASAH1 levels in TSC2-null cells. Inhibition of MEK1/2, EP3, or PKA correlated with a significant decrease of ASAH1 protein levels (Figure 4, I and J) and, with the exception of MEK1/2 inhibition, with a decrease in CREB phosphorylation (Figure 4I). Finally, estradiol (E2) led to a 2-fold increase in *ASAH1* transcript levels (Figure 4K), phosphorylation of CREB within 15 minutes (Figure 4L), and a 2.7-fold increase in ASAH1 protein levels that was concomitant with a 2.8-fold increase in ERα within 24 hours (Figure 4, M and N). Collectively, these data suggest that ASAH1 transcription and protein levels are dependent on CREB transcriptional activity.

### ASAH1 inhibitors selectively reduce the viability of TSC2-null cells.

**Figure 5:**
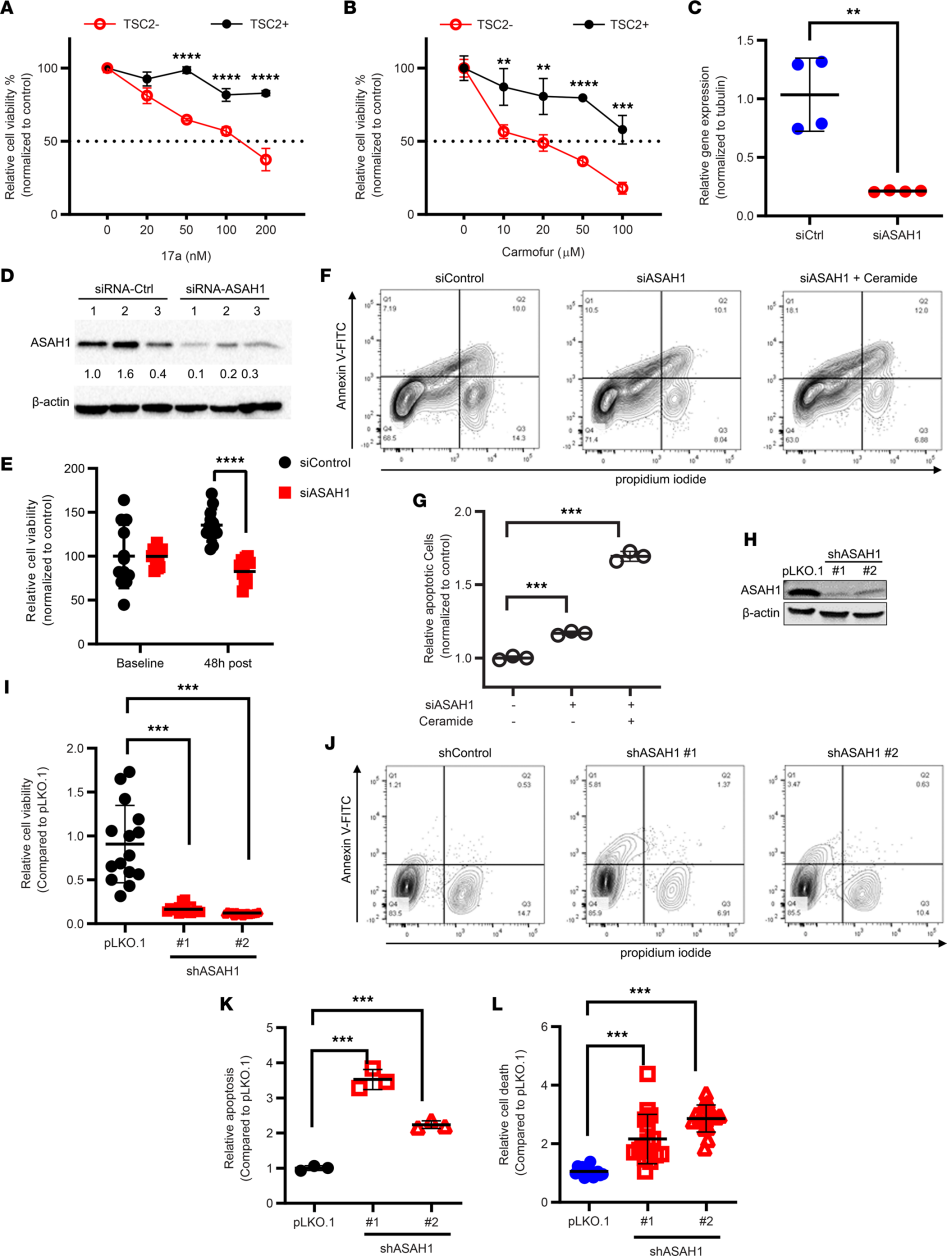
Suppression of ASAH1 decreases the survival of TSC2-null cells in vitro. 621-101 (TSC2-) and 621-103 (TSC2+) cells were treated with an ASAH1 inhibitor, 17a (**A**) or carmofur (**B**), with indicated concentrations for 72 hours. Cell viability was measured using MTT assay (*n* = 6/treatment group). Error bars show SEM. (**C**) TSC2-null 621-101 cells were transfected with 3 independent ASAH1 siRNAs or control siRNA for 48 hours. siRNA knockdown efficiency was determined by qRT-PCR (*n* = 3/group) and (**D**) immunoblotting analysis. Fold-change of ASAH1/β-actin was determined using densitometry analysis. β-Actin was used as a loading control. (**E**) Cell viability was assessed 48 hours after ASAH1 siRNA transfection in 621-101 cells using MTT assay (*n* = 12/treatment group). (**F**) Cells were treated with ceramide, then stained with Annexin V: FITC Apoptosis Detection Kit (BD). Cell death was analyzed by flow cytometry (*n* = 3). (**G**) The percentage of apoptotic (annexin V^+^) cells was determined (percentage of Annexin V: FITC-positive cells in total cell number). (**H**) 621-101 cells were infected with lentiviruses containing shRNA for vector pLKO.1, or *ASAH1*, then selected with puromycin for 2 weeks. shRNA knockdown efficiency was determined by immunoblotting. β-Actin was used as a loading control. (**I**) Viability of 621-101 cells transduced with pLKO.1 or *ASAH1* shRNA was measured by MTT assay (*n* = 8–16/treatment group). (**J**) Stable cells were harvested, then stained with Annexin V: FITC Apoptosis Detection Kit. Cell death was analyzed by flow cytometry (*n* = 3). (**K**) The percentage of apoptotic (annexin V^+^) cells was determined (percentage of Annexin V: FITC-positive cells in total cell number). (**L**) Cell death was measured using PI exclusion assay. Relative cell death was compared between pLOK.1 and shRNA-*ASAH1* cells. (**A**–**C**, **E**, **G**, **I**, **K**, and **L**) **P < 0.01, ***P < 0.001, ****P < 0.0001. (**A**–**C** and **E**) Unpaired *t* test. (**G**, **I**, **K**, and **L**) Unpaired *t* test with Bonferroni’s multiple-comparison adjustment.

To determine the effect of ASAH1 blockade on cell viability, we treated TSC2-null 621-101 cells with the ASAH1 inhibitor ARN14976 (17a) (58), which selectively decreased the viability of TSC2-null cells in a dose-dependent manner with an IC_50_ of 117 nM (Figure 5A) but not of TSC2-addback 621-103 cells (IC_50_ = 2,560 nM). Carmofur, a potent ASAH1 inhibitor that has been used to treat colorectal cancer in some countries (47, 59), selectively reduced the viability of TSC2-null cells in a dose-dependent manner (Figure 5B, IC_50_ = 17 μM) and to a lesser extent TSC2-addback cells (IC_50_ = 253 μM). These data support an important role for ASAH1 in enhancing TSC tumor cell viability.

### Molecular depletion of ASAH1 inhibits growth and induces apoptosis in AML-derived cells.

To determine whether ASAH1 is a key mediator of TSC2-null cell growth, we depleted *ASAH1* using siRNA. 621-101 cells transfected with *ASAH1* siRNA had 86.4% reduction of *ASAH1* transcript levels measured by qRT-PCR (Figure 5C) and marked reduction of ASAH1 protein levels by immunoblotting (Figure 5D). ASAH1-knockdown cells exhibited a 42% reduction in cell viability, compared with control siRNA–treated cells (Figure 5E), supporting a critical role for ASAH1 in TSC2-null cell viability. More importantly, *ASAH1* siRNA–transfected cells showed a significant increase in apoptosis, compared with control siRNA-transfected cells (Figure 5, F and G), which was further increased by ceramide treatment. Finally, we transduced TSC2-null AML-derived 621-101 cells with *ASAH1* shRNA lentiviruses or the pLKO.1 control vector (Figure 5H). Consistent with our previous results, *ASAH1* silencing resulted in significant reduction of cell viability (Figure 5I) and a significant increase in apoptosis, compared with pLKO.1-transduced cells (Figure 5, J–L). Collectively, these data support an important role for ASAH1 in TSC2-null cell survival.

### Suppression of ASAH1 attenuates the progression of xenograft tumors of TSC2-null cells.

**Figure 6:**
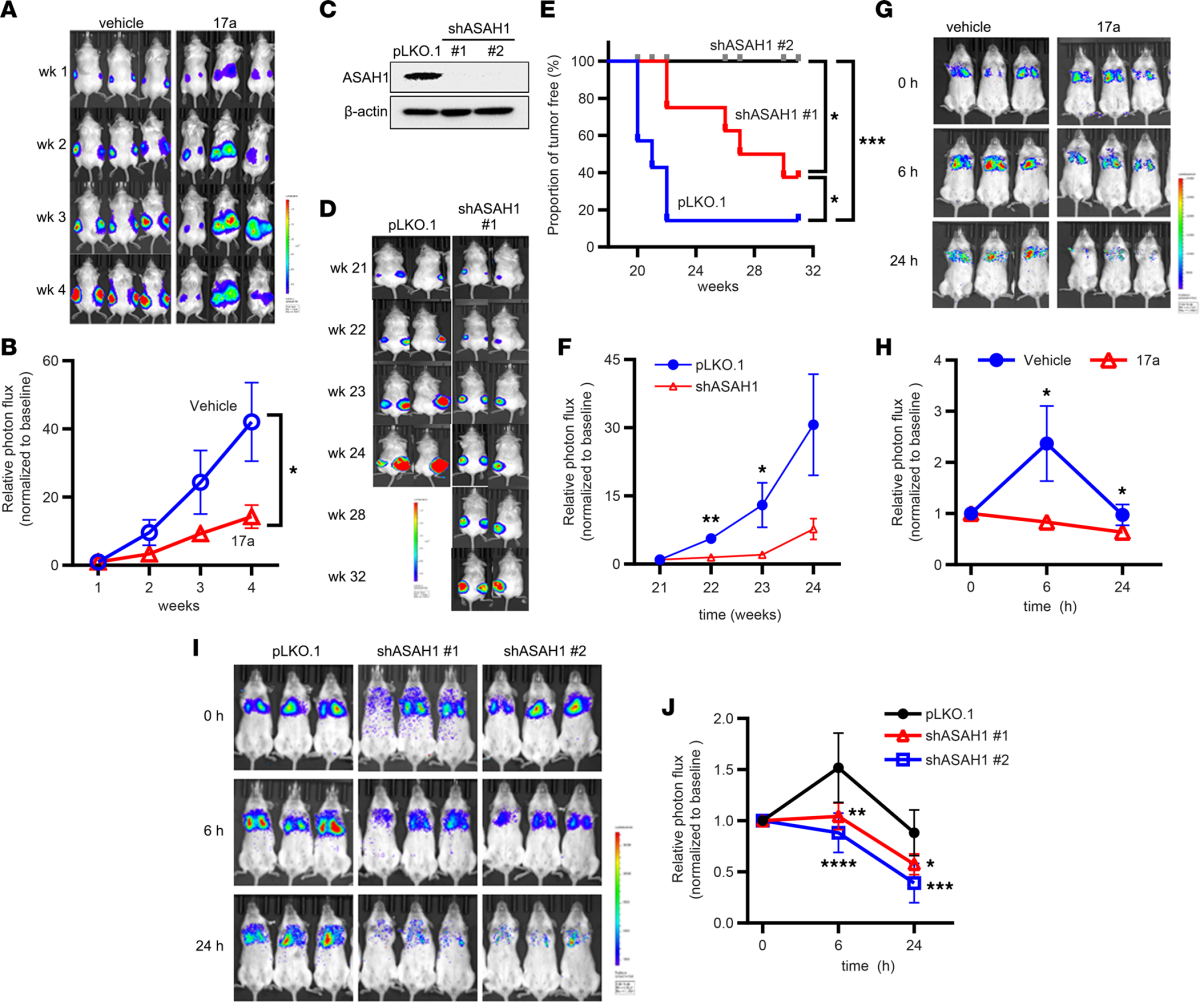
Suppression of ASAH1 decreases the survival of TSC2-null cells in vivo. (**A**) Female NSG mice were inoculated subcutaneously with 2 × 10^6^ ELT3-luciferase–expressing cells. Weekly bioluminescence imaging was performed. Upon ELT3 tumor onset, mice were randomized and treated with vehicle or 17a (10 mg/kg/day, i.p.) for 4 weeks. (**B**) Tumor photon flux was quantified and normalized to the baseline measurements (week 0). Weekly bioluminescence imaging was performed. (**C**) 621-101 cells expressing luciferase (621L9) were infected with lentivirus of ASAH1 shRNA cells or control pLKO.1 empty vector. *ASAH1* knockdown efficiency was determined by immunoblotting. β-Actin was used as a loading control. (**D**) Female NSG mice were subcutaneously inoculated with 2 × 10^6^ 621-101-pLKO.1 or 621-101-shASAH1 cells. Tumor formation was detected 21 weeks after cell inoculation. Weekly bioluminescence imaging was performed for up to 32 weeks. (**E**) Kaplan-Meier tumor-free survival curve. (**F**) Tumor photon flux was quantified and normalized to the baseline measurements (week 21) (*n* = 3–4/group). (**G**) Female NSG mice were treated with vehicle or 17a (10 mg/kg/d, i.p.) for 2 days and then intravenously inoculated with 2 × 10^5^ 621L9 cells. Bioluminescence imaging was performed 1–24 hours after cell inoculation (*n* = 3). (**H**) Photon flux at the chest region was quantified. (**I**) Female NSG mice were intravenously inoculated with 2 × 10^5^ 621L9-ASAH1-shRNA (#1 and #2) cells or control pLKO.1 cells. Bioluminescence imaging was performed 1–24 hours after cell inoculation (*n* = 4–5). (**J**) Photon flux at the chest region was quantified. (**B**, **E**, **F**, **H**, and **J**) *P < 0.05, **P < 0.01, ***P < 0.001, ****P < 0.0001. (**B**, **F**, and **H**) Unpaired *t* test. (**E**) Log-rank (Mantel-Cox) test. (**J**) Two-way ANOVA with Tukey’s multiple-comparison test.

We next assessed the possible benefit of the ASAH1 inhibitor 17a in a xenograft tumor model in which Tsc2-null ELT3 luciferase-expressing cells were implanted subcutaneously into immunodeficient NOD/SCID-γ–null (NSG) mice (25). Upon tumor development, mice were treated with 17a or vehicle control for 4 weeks. Bioluminescence imaging was performed weekly. Treatment with 17a decreased the bioluminescence intensity (Figure 6A) and significantly reduced tumor growth by 2.7-fold, relative to vehicle control (Figure 6B). To further assess the role of ASAH1 in tumor growth, LAM AML-derived TSC2-null 621-101 cells expressing luciferase were transfected with ASAH1 shRNA (Figure 6C) and inoculated in immunodeficient NSG mice. Bioluminescence signal was detectable at 21 weeks postinoculation (Figure 6D), and tumor growth was monitored until week 32 (Figure 6E). ASAH1 depletion significantly delayed the onset of tumors and decreased tumor progression by 4.2-fold, compared with pLKO.1 control–transduced cells (Figure 6, E and F).

Finally, we assessed whether suppression of ASAH1 affects the ability of TSC2-null cells to colonize the lungs in a mouse model we previously reported (25). Female NSG mice were pretreated with 17a or vehicle for 48 hours prior to intravenous injection of luciferase-expressing 621-101 cells, and bioluminescence imaging in the thoracic region was used to quantify lung colonization at baseline and at 6 and 24 hours postinjection (Figure 6G). ASAH1 blockade by 17a significantly reduced lung colonization of 621-101 cells at both time points (Figure 6H). Similarly, we inoculated NSG mice with the same number of 621-101 luciferase-expressing cells transduced with pLKO.1 control shRNA or with 2 ASAH1 shRNAs, then performed bioluminescence imaging at baseline and 6 and 24 hours postinoculation (Figure 6I). At both time points, both ASAH1 shRNA–621-101 cells exhibited significantly decreased lung colonization, compared with pLKO.1 shRNA control cells (Figure 6J). Collectively, these data support the notion that ASAH1 contributes to TSC tumor progression and dissemination to the lungs in LAM. We postulate that combinatorial suppression of mTORC1 and ASAH1 will inhibit tumor growth beyond what is achieved with mTORi alone.

### Combination of ASAH1 inhibitor 17a and rapamycin suppresses development and progression of renal cystadenomas in Tsc2-heterozygous mice.

**Figure 7:**
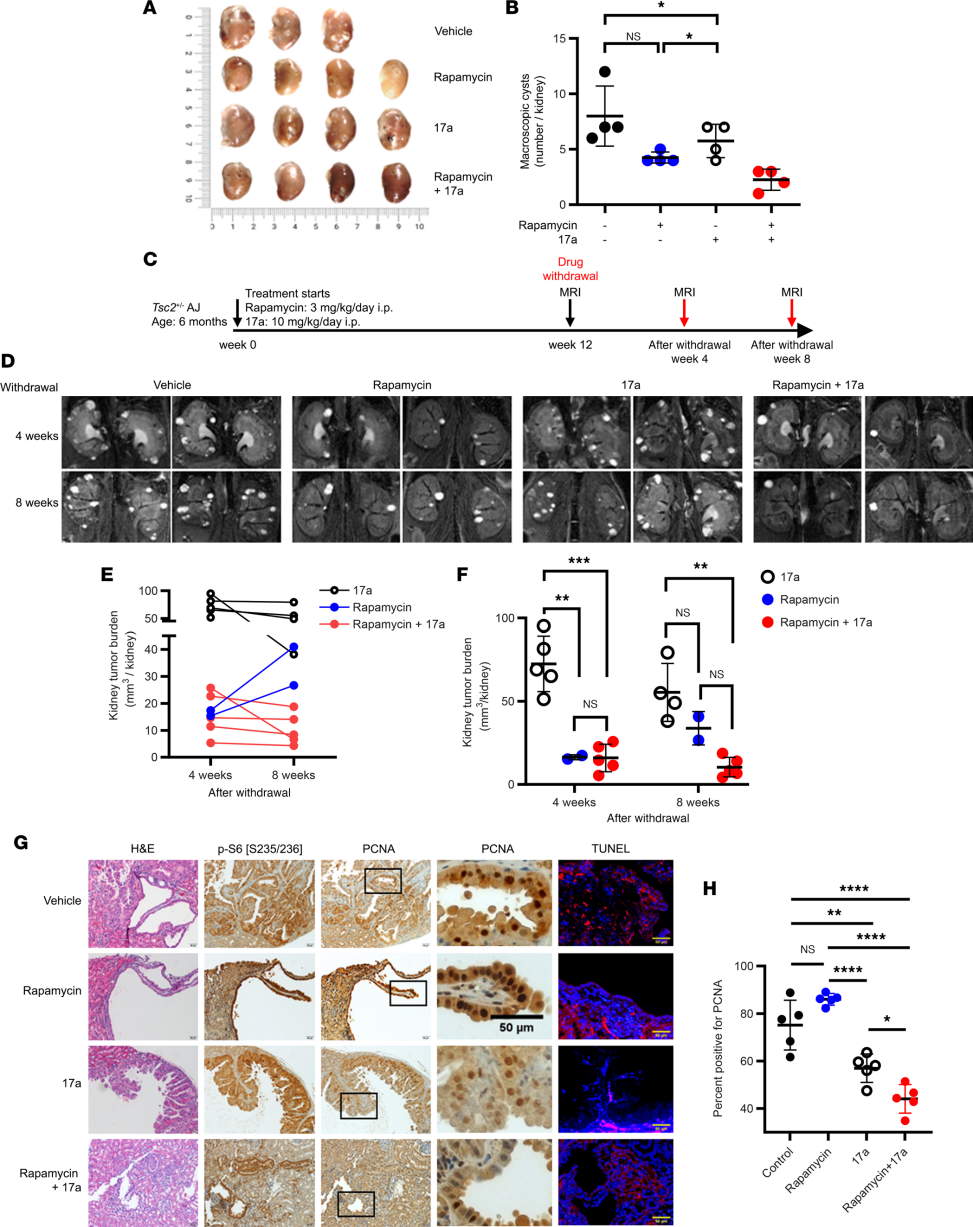
Combination of ASAH1 and mTORC1 inhibition suppresses Tsc2-null tumor growth and tumor regrowth better than rapamycin alone. *Tsc2*^+/–^ A/J mice were given vehicle, rapamycin, 17a, or rapamycin and 17a combinatorial treatment for 12 weeks (*n* = 4 mice/group). Macroscopic analysis (**A**) and quantification (**B**) of renal tumor burden under a dissection microscope upon drug withdrawal. (**C**) Tumor rebound study and MRI follow-up posttreatment schema. (**D**–**F**) MRI of *Tsc2*^+/–^ A/J mouse kidneys at 4 and 8 weeks after withdrawal from treatment. (**G**) Mouse kidney sections were stained with H&E, p-S6 (Ser235/236), PCNA, and TUNEL. Scale bars are 20 μm except for the idicated 50 μm scale bar. (**H**) Percentages of cells with nuclear immunoreactivity for PCNA were scored from 5 random fields per section. *P < 0.05, **P < 0.01, Mann-Whitney test. (**B**, **F**, and **H**) **P < 0.01, ***P < 0.001, ****P < 0.0001. (**B**) Unpaired *t* test with Bonferroni’s multiple-comparison adjustment. (**F** and **H**) One-way ANOVA with Tukey’s multiple-comparison test.

Next, we tested this combination approach in Tsc2-heterozygous mice that develop spontaneously arising renal cystadenomas. The number of macroscopic renal lesions at week 12 was markedly decreased in mice treated with 17a or rapamycin. Importantly, the combinatorial treatment of 17a and rapamycin reduced the number of renal cystadenomas to a level that was better than that achieved by either agent alone (Figure 7, A and B).

To assess the efficacy of single or combinatorial treatment on tumor rebound after drug withdrawal, all treatments were discontinued at week 12 of drug administration. Renal cystadenoma progression was monitored by MRI at 4 and 8 weeks after drug cessation (Figure 7C). MRI and quantification of individual cyst volume showed that cysts regrew within 8 weeks after withdrawal of rapamycin treatment. In contrast, renal cysts in mice withdrawn from the combinatorial treatment did not regrow, though those withdrawn from 17a treatment exhibited moderate regrowth (Figure 7, D and E). Moreover, at the end of the drug withdrawal study at 4-week and 8-week observation phases, renal lesions were counted under a dissecting microscope (60). Four weeks after drug discontinuation, the number of macroscopic renal cysts was significantly lower in mice withdrawn from rapamycin or rapamycin plus 17a treatments relative to that in mice withdrawn from 17a treatment (Figure 7F). Importantly, the combinatorial treatment of rapamycin and 17a significantly suppressed the regrowth of renal cystadenomas at the end of the 8-week observation period, compared with either agent alone (Figure 7F). Collectively, our data provide evidence that the combinatorial inhibition of mTORC1 and ASAH1 suppresses the progression and regrowth of renal cystadenomas after drug cessation in vivo.

To assess the mechanisms for 17a-dependent delay in posttreatment regrowth of renal cysts/cystadenomas, we performed immunohistochemical staining of p-S6 (Ser235/236) and proliferating cell nuclear antigen (PCNA), as well as TUNEL assay, in kidneys from mice withdrawn from treatments. We found that cells lining renal cysts and in renal lesions were positive for p-S6 (Figure 7G), indicative of mTORC1 hyperactivation. TUNEL staining was negative in kidneys from mice in all groups (Figure 7G), suggesting either that treatments did not induce apoptosis in cystadenoma lesions or that apoptotic cystadenoma cells were rapidly cleared after drug withdrawal. Quantification of PCNA immunoreactivity in tumors after drug withdrawal showed a significant reduction in PCNA nuclear positivity for the 17a alone and rapamycin plus 17a combinatorial treatment groups, compared with vehicle- and rapamycin-only treatment groups (Figure 7, G and H). These data suggest that the 17a-dependent delay in posttreatment regrowth is due to a reduction in TSC2-null tumor cell proliferation.

## Discussion

Sphingolipids play critical roles in kidney physiology (61), chronic inflammation, and cancer progression (39, 40). Various sphingolipids have widely differing cellular actions, and we postulated that they may affect renal AML–derived cell viability. In this study, we show that the increased growth of TSC and LAM tumors is, at least in part, attributed to the aberrant expression of DEGS1 and ASAH1 resulting in changes in sphingolipid levels. We found that renal AMLs accumulate abundant levels of DEGS1 and ASAH1 and that ASAH1 suppression leads to reduced viability and increased apoptosis of TSC2-null cells. Furthermore, TSC2 negatively regulates ASAH1 expression and sphingosine production in an mTORi-independent manner.

Our data show that rapamycin suppresses mTORC1 activation, as assessed by p-S6 levels, but has no effect on ASAH1 expression or activity in TSC2-null cells. There are several potential explanations for this finding. Studies by Choo et al. have shown that rapamycin’s inhibition of mTORC1 is incomplete (62), and mTORC2 dysfunction has been observed in TSC2-null cells (63). There is further evidence that TSC2 may have functions in addition to inhibiting mTORC1 or mTORC2. Here, we sought to determine the mechanism by which TSC2 regulates the expression of sphingolipid biosynthesis genes and found that cAMP-CREB mediates the ASAH1 expression in TSC2-null cells.

It is important to note that mTORi act principally on mTORC1 rather than mTORC2, even though TSC mutations affect both complexes. Logically, the mTORC2-mediated pathways likely contribute to mTORC1-independent cellular behaviors in the presence of TSC mutations or mTORi treatment (3). Notably, B-Raf kinase activity is reduced in TSC2-null cells because of an mTORC1-independent action of Rheb (64, 65). *Tsc1*^–/–^ and *Tsc2*^–/–^ mouse embryonic fibroblasts (MEFs) have a higher percentage of ciliated cells compared with controls, and the mTORi rapamycin has no effect on the abundance of cilia (66). A recent study suggests that sphingolipid metabolism, a well-known target of mTORC2-mediated pathways, may be of importance (67).

We, and others, have previously shown that estrogen promotes MAPK phosphorylation in TSC2-null cells in vitro (25, 35, 68–71) and in vivo (25). We also reported that estrogen increased prostaglandin biosynthesis (35) and functions via prostaglandin receptor EP3 (36). In the present study, we found TSC2-null cells exhibited higher cellular levels of cAMP, phosphorylation of CREB, and nuclear localization of p-CREB (Figure 4, F and G), indicating the activation of cAMP-CREB. We also found that E2 treatment increased the transcript and protein levels of ASAH1 in TSC2-null cells (Figure 4, K and L). Importantly, elevated protein levels of ASAH1 were attenuated by an MEK-MAPK inhibitor (AZD6244), an EP3 antagonist (L798106), and a PKI (Figure 4I), suggesting the action of MAPK and PKA in enhancing ASAH1 expression in TSC2-null cells. It has been reported that CREB is a transcription factor mediating ASAH1 expression in human adrenocortical cells (54). PKA and MAPK also activate CREB (57). Thus, our current study reveals a connection among estrogen, MAPK, prostaglandin action, cAMP-CREB, and ASAH1 expression in TSC2-null cells, consistent with other reported findings.

Renal AMLs are a major clinical manifestation of TSC. These tumors are composed of aberrant blood vessels, smooth muscle cells, and adipose cells (53). We found increased DEGS1 and ASAH1 protein levels in renal AML cells from 3 patients. We have shown that TSC renal AMLs express abundant DEGS1 and ASAH1 proteins relative to adjacent normal kidney tissues. Treatment with rapamycin alone has a cytostatic rather than remission-inducing effect in clinical trials and in preclinical models of TSC. These data suggest that, within the context of mTORi actions, there are primary pathways provoked by TSC mutations that promote persistent proliferative states. Our studies show that suppression of ASAH1 using 17a and ASAH1 shRNA attenuates xenograft tumor progression and growth of renal cystadenomas in *Tsc2*^+/–^ A/J mice. Our data support the concept that the selectively increased expression of DEGS1 and ASAH1 in TSC tumor cells leads to increased sphingolipid biosynthesis, which in turn enhances TSC tumor cell viability and growth in preclinical models. Interestingly, studies have shown that *Tsc2*^–/–^ MEFs are resistant to ceramide-triggered death (72). Collectively, these results suggest a cell context–specific regulation of sphingolipid metabolism and actions in TSC.

In summary, the present study identified increased expression of key enzymes in the sphingolipid biosynthesis pathway in LAM and AML tumor cells as well as in TSC2-null cells. The activation of the sphingolipid biosynthesis pathway in TSC-deficient cells and tumors is rapamycin insensitive. We further showed that TSC2 regulates ASAH1 to increase sphingolipid production and activity. Thus, we have identified the sphingolipid biosynthesis pathway as a promising therapeutic target with potential clinical value in TSC and LAM and possibly in other mTORC1-hyperactive neoplasms.

## Methods

### scRNA-Seq analysis.

LAM lung tissue collection, scRNA-Seq data analyses, and cell type annotation were described in detail in Guo et al., 2020 (52). In the present study, we integrated scRNA-Seq of LAM lungs (*n* = 2, GEO GSE135851), control female lung samples (*n* = 6, GEO GSE122960), and renal AML (*n* = 1, GEO GSE135851/GSM4035469). Cell type annotation was done based on the previous study (52). For LAM lungs and control lungs, a total of 54,511 cells were clustered and dissected into major lineages based on lineage pan-markers (epithelial: EPCAM, CDH1; endothelial: PECAM1, CDH5; mesenchymal: TCF21, ACTA2; immune: PTPRC, CD68). Differentially expressed genes between LAM^CORE^ cells (52) and control mesenchymal cells were identified using Wilcoxon’s signed-rank test. Functional enrichment analysis was performed using ToppGene suite (73).

### Cell culture, reagents, and assays.

Eker rat uterine leiomyoma–derived (ELT3) cells (74, 75) were provided by C. Walker (Institute of Biosciences and Technology, Texas A&M University, Houston, Texas, USA). ELT3 cells stably expressing luciferase (ERL4) and LAM patient–associated AML–derived 621-101 cells were provided by E.P. Henske (Brigham and Women’s Hospital and Harvard Medical School, Boston, Massachusetts, USA) (25, 76). Cells were cultured in DMEM/F12 supplemented with 10% FBS and 1% penicillin-streptomycin (Gibco). For immunoblotting, cells or tissues were lysed in M-PER or T-PER buffer (Thermo Fisher Scientific) supplemented with protease and phosphatase inhibitors (MilliporeSigma). Protein was quantified using Bradford assay (Bio-Rad), and equal amounts of protein were separated by gel electrophoresis (Bio-Rad) and transferred onto PVDF membranes (MilliporeSigma). Membranes were probed with antibodies against p-CREB (catalog 9198), CREB (catalog 9197), p-S6 (catalog 4858), TSC2 (catalog 4308), 4E-BP1 (catalog 9452), p-ERK1/2 (catalog 4370), and β-actin (catalog 4967) from Cell Signaling Technology; ASAH1 (catalog 602302) from BD Transduction Lab; DEGS1 (catalog ab167169) from Abcam; and ERα (H184) (catalog sc-7207) from Santa Cruz Biotechnology, then incubated with secondary HRP-tagged antibodies (Amersham, NA931 or NA934). Blots were incubated using chemiluminescent reagents (Thermo Fisher Scientific) and developed using G:BOX Imaging System (Discovery Scientific Solutions). Uncropped gel images are included in the published online supplemental material.

### Confocal microscopy.

ELT3 and LAM-derived cells were plated overnight on glass coverslips in 12-well tissue culture plates. Cells were serum-starved overnight, then treated with 20 nM rapamycin for 24 hours. Cells were rinsed with PBS twice, fixed with 4% paraformaldehyde for 30 minutes at 37°C, permeabilized with 0.2% Triton X-100, blocked in 3% BSA/PBS for 1 hour, and then incubated with primary antibody p-CREB (S133) (catalog 9198 from Cell Signaling Technology) in 1% BSA/PBS for 1 hour followed by secondary antibody donkey anti–rabbit IgG (H+L) (catalog A10042 from Invitrogen) for 1 hour at room temperature. Images were captured with a FluoView FV-10i Olympus laser point-scanning confocal microscope.

### Expression array analysis.

Reanalysis of previously published expression array data (GEO GSE16944) (51) was performed using GEO2R online tool (NIH). Transcript levels were compared between TSC2-deficient (TSC2^–^) and TSC2-addback (TSC2^+^) cells or rapamycin-treated and vehicle-treated TSC2-deficient (TSC2^–^) cells.

### siRNA transfections.

Two separate human ASAH1 siRNAs (50 nM) (Dharmacon) were transfected into 621-101 cells using Lipofectamine RNAiMAX (Invitrogen) according to the manufacturer’s protocols. Cells were harvested 48 hours posttransfection.

### shRNA downregulation.

HEK293T (ATCC) packaging cells were transfected with *ASAH1* shRNA or nontargeting shRNA vectors using *Trans*IT TKO Transfection Reagent (Mirus). 621-101 cells were transduced with lentiviruses for 48 hours and then selected with puromycin. Stable clones were harvested for future experiments.

### qRT-PCR.

RNA from cultured cells was isolated using RNeasy Mini Kit (QIAGEN). Gene expression was quantified using One-Step qRT-PCR Kits (Invitrogen) in the Applied Biosystems Step One Plus Real-Time PCR System and normalized to *ACTB* (human β-actin) or *Tuba1a* (rat α-tubulin). PCR primer sequences are *ASAH1* forward 5′ CTTTGCTGGCTATGTGGGCATG 3′ and reverse 5′ TGAGGAACCCTATCCACATGGC 3′ and *ACTB* forward 5′ AGAGCCTCGCCTTTGCCG 3′ and reverse 5′ CCCACGATGGAGGGGAAGAC 3′.

### Immunofluorescence staining.

Sections were deparaffinized, incubated with primary antibody (1:100 in PBS+3%BSA) smooth muscle actin (1:200 in PBS+3% BSA, Santa Cruz Biotechnology, sc32251) and secondary antibodies (1:1,000, Invitrogen, A-21202 and A10042). Images were captured with a fluorescence microscope (Olympus BX60).

### FACS.

621-101 cells were stained with Annexin V-FITC Apoptosis Detection Kit I (BD 556547) according to the manufacturer’s protocols. FACS analysis was carried out on BD FACSCanto II.

### LC/MS-MS profiling of sphingolipids.

Acetonitrile, methanol, isopropanol, ethyl acetate, and formic acid (99%) were purchased from Thermo Fisher Scientific. Ammonium formate was analytical grade and purchased from MilliporeSigma. Sphingosine (d17:1), ceramide (d18:1/17:0), sphingosine (d18:1), ceramide (d18:1/16:0), ceramide (d18:1/18:0), and ceramide (d18:1/24:0) were purchased from Avanti Polar Lipids. Calibration standards containing 0, 0.1, 1, 5, 10, and 20 ng/mL of sphingosine and 0, 10, 100, 500, 1,000, and 2,000 ng/mL ceramides, including ceramide (d18:1/16:0), ceramide (d18:1/18:0), ceramide (d18:1/24:0), were prepared by pipetting the appropriate volume of standards into the charcoal-stripped human serum (Equitech-Bio, Inc.). Calibrators were extracted using the same procedure as the plasma samples. Quantification of sphingolipid species was based on respective calibration curves from sphingosines and ceramides. Plasma samples were stored at −80°C and allowed to thaw at 4°C for 2 hours. Extraction of sphingolipids from plasma samples (25 μL) was conducted with a modified method based on Hammad et al. (77). Briefly, deionized water (950 μL) was first added to each sample, which was also fortified with 25 μL of appropriate internal standards (appropriate concentration of C17-sphingosine and C17-ceramide in MeOH). The samples were then extracted with 1 mL extraction solution consisting of isopropanol and ethyl acetate (15:85 v/v). After centrifugation for 5 minutes at 3,220×*g* at room temperature, the upper organic phase was collected. The remaining diluted plasma was then acidified with 50 μL formic acid (98%), and an additional 1 mL of extraction solution was added to further facilitate completion of extraction. The organic phase was then combined and was evaporated to dryness. The dried residues were reconstituted in 150 μL methanol and transferred to HPLC auto-sampler vials (Waters) for UPLC-MS/MS analysis. For ceramide analysis, extraction samples were further diluted 10 times with methanol due to their high concentrations. Quantification of sphingolipids was performed by a UHPLC system coupled to a triple-quadrupole mass spectrometer (Waters). Multiple reaction monitoring (MRM) mode was used for quantification of sphingolipids, and chromatography was conducted on an Acquity UHPLC BEH C18 column (2.1 × 100 mm, 1.7 μm, Waters). The optimal signal for the ion pairs of sphingosine and ceramides was achieved in positive ion mode with the following instrument settings: capillary voltage, 2.0 kV; cone voltage 10 V; desolvation temperature, 400°C; desolvation gas flow, 900 L/h; and cone gas flow, 10 L/h. Helium was used as the collision gas. A gradient mobile phase was used with a binary solvent system, which ramped from 40% solvent B to 100% solvent B over 4 minutes, after which it was held for 5 minutes, then changed to 40% solvent B over 0.1 minute before being held for 2.9 minutes for re-equilibration. The total run time was 10 minutes using a flow rate at 0.4 mL/min. Solvent A consisted of water/methanol (95/5 v/v) with 10 mM ammonium formate and 0.1% formic acid; solvent B consisted of methanol with 10 mM ammonium formate and 0.1% formic acid. Details of MRM transitions monitored, cone and collision voltages, and retention times for analytes and internal standards are shown in Table 1 and Table 2. Data were acquired and processed with MassLynx 4.1 software (Waters).

### Cell viability assay.

Cell viability was estimated using the MTT assay. TSC2-null 621-101 and TSC2-addback 621-103 cells were plated in 96-well plates (2 × 10^3^ cells/well). Cells were incubated in a 37°C CO_2_ incubator for 24 hours before treatment with escalating concentrations of 17a (Cayman Chemical 17119) or carmofur (Selleck Chemicals S1289) for 72 hours. A total of 25 μL of MTT solution (2.5 mg/mL growth media) was added to each well, followed by 4 hours’ incubation at 37°C. Formation of formazan crystals was observed under the microscope. At the end of treatment, an equal volume of 0.04N HCl in isopropanol was added, followed by 1.5 hours’ incubation of plates at 37°C. Absorbance at 560 nm was measured in a Synergy HTX multimode reader (Biotek) with a reference of 650 nm. The concentration of the drug that reduces total growth by 50% was calculated using CompuSyn software (ComboSyn, Inc.) (78).

### Immunohistochemistry.

Sections were deparaffinized, incubated with primary antibodies — p-S6 (S235/236) (4857S), PCNA (2586S), and TUNEL (48513) from Cell Signaling Technology; ASAH1 (602302) from BD Transduction Lab; and ACTA2 (ab5694) and DEGS1 (ab167169) from Abcam — and biotinylated secondary antibodies — Mouse and Rabbit Specific HRP/DAB (ABC) Detection IHC kit (ab64264 from Abcam) — and counterstained with Gill’s hematoxylin. Cell death was assessed using TUNEL assay (Cell Signaling Technology).

### Drug formulation for in vivo studies.

Two hours prior to treatment, rapamycin (Enzo) was diluted in 10% PEG300 and 0.5% Tween 80 in PBS (Corning). 17a (Cayman Chemical) was diluted in PBS (Corning).

### Animal studies.

For ELT3-based studies, female C.B-*Igh*-*1^b^*/IcrTac-*Prkdc^scid^* (SCID) mice at 4–6 weeks of age were purchased from Taconic Biosciences. For 621-101–based studies, female NOD.Cg-*Prkdc*^scid^ *Il2rg*^tm1Wjl^/*SzJ* (NSG) mice at 7–8 weeks of age were purchased from The Jackson Laboratory. Animal health was monitored daily during studies. Animal weight was monitored weekly.

For xenograft studies, 2 × 10^6^ ELT3-luciferase or 10^7^ 621-101–luciferase cells were resuspended in PBS with 25% Matrigel (BD) and injected subcutaneously into the flanks of mice as previously described (25). Upon tumor onset, mice were randomized into 2 groups: vehicle and 17a (10 mg/kg/d, i.p.) (49). Treatments were given 5 times per week for 4 weeks (or less time, if necessary, because of animal pain or distress).

For short-term lung colonization studies, NSG mice were pretreated with vehicle or 17a (10 mg/kg/d, i.p.) for 2 days before cell inoculation. 2 × 10^5^ 621-101–luciferase cells were resuspended in 0.1 mL PBS and injected into mice intravenously as previously described (25).

For renal cystadenoma studies, *Tsc2*^+/–^ A/J mice at 9 months old were obtained as a gift from Steve Roberds (Tuberous Sclerosis Alliance, Washington, DC, USA). *Tsc*2^+/–^ A/J mice develop renal cystadenomas at high frequencies (79, 80). Four treatment groups were studied, including 1 vehicle control group. Mice were treated at 5 months of age with rapamycin (3 mg/kg/d, i.p.), 17a (10 mg/kg/d, i.p.), and rapamycin plus 17a (3 mg/kg/d, i.p. + 10 mg/kg/d, i.p.). Treatments were given 5 times per week. Eight animals were treated for 4 months and then sacrificed at 9 months of age. To examine tumor regrowth, 8 mice were withdrawn from treatment for 2 months and then sacrificed at 11 months of age. Renal tumor burden was assessed by MRI every 4 weeks.

### Bioluminescent reporter imaging.

Ten minutes before imaging, mice were given d-luciferin (120 mg/kg, i.p., PerkinElmer Inc., 122799). Bioluminescent signals were recorded using the Xenogen IVIS Spectrum System. Total photon flux of chest regions was analyzed as previously described (25).

### T2-weighted MRI to assess renal tumor progression in Tsc2^+/–^ mice.

MRI scans were carried out by the In vivo Microimaging Laboratory at Cincinnati Children’s Hospital Medical Center using a 9.4 T vertical-bore Bruker Biospec system using a 36 mm diameter volume transmit/receive coil (81). Mice were anesthetized with isoflurane and positioned in the center of the coil and magnet. Fat-suppressed, respiratory triggered, 2D, fast spin echo data were acquired using a repetition time of 3,400 ms, an echo time of 42 ms, 4 averages, an echo spacing of 14 ms with 8 echoes acquired per repetition time, a matrix of 256 × 256 with a 29.5 mm × 29.5 mm field of view with a 50 kHz bandwidth, 0.5 mm slice thickness, and 19 slices to cover kidney.

### Statistics.

Unless otherwise specified, data in graphs represent mean ± SD. Statistical analyses and plotting were performed using GraphPad Prism version 9.5.1, including unpaired *t* test and Mann-Whitney test when comparing 2 groups for in vitro and in vivo studies, unpaired *t* test with Bonferroni’s multiple-comparison adjustments, and 1- and 2-way ANOVA tests for multiple-group comparison (Dunnett’s multiple-comparison test when comparing multiple groups with control group, Tukey’s multiple-comparison test when making multiple pair-wise comparisons between different groups). A *P* value less than 0.05 was considered significant.

### Study approval.

The University of Cincinnati Standing Committees on Animals approved all procedures described according to standards as outlined in the *Guide for the Care and Use of Laboratory Animals* (National Academies Press, 2011) (UC-IACUC 21-06-01-01). The Institutional Review Board of the University of Cincinnati approved all human-relevant studies (UC-IRB 2020-0097).

## Author contributions

YX and JJY conceived and designed the study. CL and EYZ conducted experiments and acquired data. AGZ performed quantification of immunoblotting and immunohistochemical staining. TO performed immunoblotting analysis for revision. LP performed TUNEL staining in mouse tissues. RJ supervised statistical analysis. XZ, RH, and KDRS performed lipidomic analysis. KAWB performed quality control and selection of LAM tissues for scRNA-Seq. MMM and MK analyzed and interpreted immunohistochemical staining with ASAH1 and DEGS1 in renal AMLs and healthy kidney tissues. EF and DL conducted mouse MRI and data analysis. SZ, MG, and YX analyzed scRNA-Seq data. EJK, NG, KAWB, and FXM assisted with specimen acquisition, data interpretation, and clinical context. AA, UM, and EYZ analyzed data. AA and EPS, YX, and JJY wrote the manuscript. All authors reviewed the final version of the manuscript. AA, CL, and EYZ contributed equally, have the right to list their name first in their CV, and should be considered co–first authors. The order of co–first authors was determined based on intellectual contribution and volume of work.

## Supplementary Material

Supplemental data

## Figures and Tables

**Table 2:**
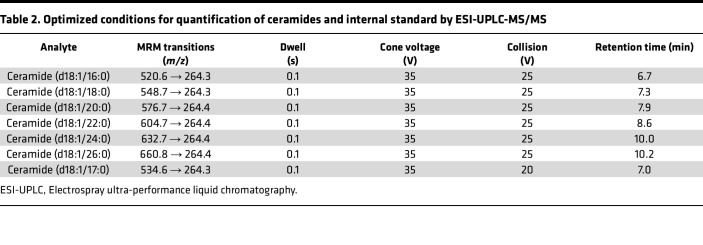
Optimized conditions for quantification of ceramides and internal standard by ESI-UPLC-MS/MS

**Table 1:**
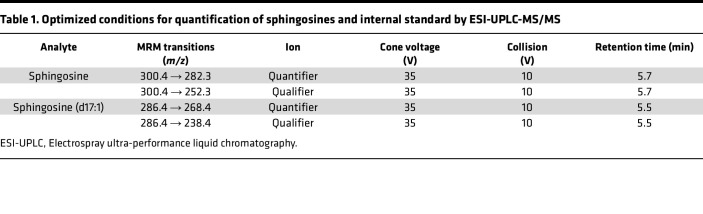
Optimized conditions for quantification of sphingosines and internal standard by ESI-UPLC-MS/MS

## Version 1 03/16/2023

In-Press Preview

## Version 2 05/08/2023

Electronic publication

## References

[1] Crino PB (2006). The tuberous sclerosis complex. N Engl J Med.

[2] Henske EP (2016). Tuberous sclerosis complex. Nat Rev Dis Primers.

[3] Neuman NA, Henske EP (2011). Non-canonical functions of the tuberous sclerosis complex-Rheb signalling axis. EMBO Mol Med.

[4] https://www.uptodate.com/contents/sporadic-lymphangioleiomyomatosis-clinical-presentation-and-diagnostic-evaluation#.

[5] Cudzilo CJ (2013). Lymphangioleiomyomatosis screening in women with tuberous sclerosis. Chest.

[6] Kristof AS (2015). Lymphangioleiomyomatosis and tuberous sclerosis complex in Quebec: prevalence and health-care utilization. Chest.

[7] Adriaensen ME (2011). Radiological evidence of lymphangioleiomyomatosis in female and male patients with tuberous sclerosis complex. Clin Radiol.

[8] Ryu JH (2012). Cystic lung disease is not uncommon in men with tuberous sclerosis complex. Respir Med.

[9] Carsillo T (2000). Mutations in the tuberous sclerosis complex gene TSC2 are a cause of sporadic pulmonary lymphangioleiomyomatosis. Proc Natl Acad Sci U S A.

[10] Giannikou K (2016). Whole exome sequencing identifies TSC1/TSC2 biallelic loss as the primary and sufficient driver event for renal angiomyolipoma development. PLoS Genet.

[11] Strizheva GD (2001). The spectrum of mutations in TSC1 and TSC2 in women with tuberous sclerosis and lymphangiomyomatosis. Am J Respir Crit Care Med.

[12] Castro AF (2003). Rheb binds tuberous sclerosis complex 2 (TSC2) and promotes S6 kinase activation in a rapamycin- and farnesylation-dependent manner. J Biol Chem.

[13] Duvel K (2010). Activation of a metabolic gene regulatory network downstream of mTOR complex 1. Mol Cell.

[14] Inoki K (2003). Rheb GTPase is a direct target of TSC2 GAP activity and regulates mTOR signaling. Genes Dev.

[15] Zoncu R (2011). mTOR: from growth signal integration to cancer, diabetes and ageing. Nat Rev Mol Cell Biol.

[16] Hay N, Sonenberg N (2004). Upstream and downstream of mTOR. Genes Dev.

[17] Menon S, Manning BD (2008). Common corruption of the mTOR signaling network in human tumors. Oncogene.

[18] Schmelzle T, Hall MN (2000). TOR, a central controller of cell growth. Cell.

[19] Sarbassov DD (2004). Rictor, a novel binding partner of mTOR, defines a rapamycin-insensitive and raptor-independent pathway that regulates the cytoskeleton. Curr Biol.

[20] El-Hashemite N (2003). Loss of Tsc1 or Tsc2 induces vascular endothelial growth factor production through mammalian target of rapamycin. Cancer Res.

[21] Karbowniczek M (2010). The evolutionarily conserved TSC/Rheb pathway activates Notch in tuberous sclerosis complex and Drosophila external sensory organ development. J Clin Invest.

[22] Kenerson HL (2002). Activated mammalian target of rapamycin pathway in the pathogenesis of tuberous sclerosis complex renal tumors. Cancer Res.

[23] Lee L (2005). Efficacy of a rapamycin analog (CCI-779) and IFN-gamma in tuberous sclerosis mouse models. Genes Chromosomes Cancer.

[24] Parkhitko A (2011). Tumorigenesis in tuberous sclerosis complex is autophagy and p62/sequestosome 1 (SQSTM1)-dependent. Proc Natl Acad Sci U S A.

[25] Yu JJ (2009). Estrogen promotes the survival and pulmonary metastasis of tuberin-null cells. Proc Natl Acad Sci U S A.

[26] Bissler JJ (2008). Sirolimus for angiomyolipoma in tuberous sclerosis complex or lymphangioleiomyomatosis. N Engl J Med.

[27] Bissler JJ (2013). Everolimus for angiomyolipoma associated with tuberous sclerosis complex or sporadic lymphangioleiomyomatosis (EXIST-2): a multicentre, randomised, double-blind, placebo-controlled trial. Lancet.

[28] McCormack FX (2011). Efficacy and safety of sirolimus in lymphangioleiomyomatosis. N Engl J Med.

[29] Franz DN (2013). Efficacy and safety of everolimus for subependymal giant cell astrocytomas associated with tuberous sclerosis complex (EXIST-1): a multicentre, randomised, placebo-controlled phase 3 trial. Lancet.

[30] Taveira-DaSilva AM (2011). Changes in lung function and chylous effusions in patients with lymphangioleiomyomatosis treated with sirolimus. Ann Intern Med.

[31] Davies DM (2011). Sirolimus therapy for angiomyolipoma in tuberous sclerosis and sporadic lymphangioleiomyomatosis: a phase 2 trial. Clin Cancer Res.

[32] Kwiatkowski DJ (2010). Animal models of lymphangioleiomyomatosis (LAM) and tuberous sclerosis complex (TSC). Lymphat Res Biol.

[33] Yu J, Henske EP (2010). mTOR activation, lymphangiogenesis, and estrogen-mediated cell survival: the “perfect storm” of pro-metastatic factors in LAM pathogenesis. Lymphat Res Biol.

[34] Franz DN (2006). Rapamycin causes regression of astrocytomas in tuberous sclerosis complex. Ann Neurol.

[35] Li C (2014). Estradiol and mTORC2 cooperate to enhance prostaglandin biosynthesis and tumorigenesis in TSC2-deficient LAM cells. J Exp Med.

[36] Li C (2017). Tuberin regulates prostaglandin receptor-mediated viability, via Rheb, in mTORC1-hyperactive cells. Mol Cancer Res.

[37] Li C (2014). Rapamycin-insensitive up-regulation of adipocyte phospholipase A2 in tuberous sclerosis and lymphangioleiomyomatosis. PLoS One.

[38] Priolo C (2015). Tuberous sclerosis complex 2 loss increases lysophosphatidylcholine synthesis in lymphangioleiomyomatosis. Am J Respir Cell Mol Biol.

[39] Wang D, Dubois RN (2010). Eicosanoids and cancer. Nat Rev Cancer.

[40] Wang MT (2007). Cyclooxygenases, prostanoids, and tumor progression. Cancer Metastasis Rev.

[41] De Keijzer S (2013). The multiple faces of prostaglandin E2 G-protein coupled receptor signaling during the dendritic cell life cycle. Int J Mol Sci.

[42] Lahiri S, Futerman AH (2007). The metabolism and function of sphingolipids and glycosphingolipids. Cell Mol Life Sci.

[43] Ogretmen B (2018). Sphingolipid metabolism in cancer signalling and therapy. Nat Rev Cancer.

[44] Ghering AB, Davidson WS (2006). Ceramide structural features required to stimulate ABCA1-mediated cholesterol efflux to apolipoprotein A-I. J Lipid Res.

[45] Witting SR (2003). Ceramide enhances cholesterol efflux to apolipoprotein A-I by increasing the cell surface presence of ATP-binding cassette transporter A1. J Biol Chem.

[46] Ogretmen B, Hannun YA (2004). Biologically active sphingolipids in cancer pathogenesis and treatment. Nat Rev Cancer.

[47] Realini N (2013). Discovery of highly potent acid ceramidase inhibitors with in vitro tumor chemosensitizing activity. Sci Rep.

[48] Nakamura T (2001). Optimal duration of oral adjuvant chemotherapy with Carmofur in the colorectal cancer patients: the Kansai Carmofur Study Group trial III. Int J Oncol.

[49] Doan NB (2017). Acid ceramidase and its inhibitors: a de novo drug target and a new class of drugs for killing glioblastoma cancer stem cells with high efficiency. Oncotarget.

[50] Islam MM, Mirza SP (2022). Versatile use of Carmofur: a comprehensive review of its chemistry and pharmacology. Drug Dev Res.

[51] Lee PS (2010). Rapamycin-insensitive up-regulation of MMP2 and other genes in tuberous sclerosis complex 2-deficient lymphangioleiomyomatosis-like cells. Am J Respir Cell Mol Biol.

[52] Guo M (2020). Single-cell transcriptomic analysis identifies a unique pulmonary lymphangioleiomyomatosis cell. Am J Respir Crit Care Med.

[53] Karbowniczek M (2003). Renal angiomyolipomas from patients with sporadic lymphangiomyomatosis contain both neoplastic and non-neoplastic vascular structures. Am J Pathol.

[54] Lucki N, Sewer MB (2009). The cAMP-responsive element binding protein (CREB) regulates the expression of acid ceramidase (ASAH1) in H295R human adrenocortical cells. Biochim Biophys Acta.

[55] Boyle SC (2014). Notch signaling is required for the formation of mesangial cells from a stromal mesenchyme precursor during kidney development. Development.

[56] Xie J (2011). cAMP inhibits mammalian target of rapamycin complex-1 and -2 (mTORC1 and 2) by promoting complex dissociation and inhibiting mTOR kinase activity. Cell Signal.

[57] Barlow CA (2007). Asbestos-mediated CREB phosphorylation is regulated by protein kinase A and extracellular signal-regulated kinases 1/2. Am J Physiol Lung Cell Mol Physiol.

[58] Pizzirani D (2015). Benzoxazolone carboxamides: potent and systemically active inhibitors of intracellular acid ceramidase. Angew Chem Int Ed Engl.

[59] Ito K (1996). Oral adjuvant chemotherapy with carmofur (HCFU) for colorectal cancer: five-year follow-up. Tokai HCFU Study Group--third study on colorectal cancer. J Surg Oncol.

[60] Pollizzi K (2009). Equivalent benefit of mTORC1 blockade and combined PI3K-mTOR blockade in a mouse model of tuberous sclerosis. Mol Cancer.

[61] Koch A (2013). Sphingosine 1-phosphate in renal diseases. Cell Physiol Biochem.

[62] Choo AY (2010). Glucose addiction of TSC null cells is caused by failed mTORC1-dependent balancing of metabolic demand with supply. Mol Cell.

[63] Huang J (2008). The TSC1-TSC2 complex is required for proper activation of mTOR complex 2. Mol Cell Biol.

[64] Karbowniczek M (2004). Regulation of B-Raf kinase activity by tuberin and Rheb is mammalian target of rapamycin (mTOR)-independent. J Biol Chem.

[65] Karbowniczek M (2006). Rheb inhibits C-raf activity and B-raf/C-raf heterodimerization. J Biol Chem.

[66] Hartman TR (2009). The tuberous sclerosis proteins regulate formation of the primary cilium via a rapamycin-insensitive and polycystin 1-independent pathway. Hum Mol Genet.

[67] Guri Y (2017). mTORC2 promotes tumorigenesis via lipid synthesis. Cancer Cell.

[68] Gu X (2013). Integration of mTOR and estrogen-ERK2 signaling in lymphangioleiomyomatosis pathogenesis. Proc Natl Acad Sci U S A.

[69] Sun Y (2014). Progesterone and estradiol synergistically promote the lung metastasis of tuberin-deficient cells in a preclinical model of lymphangioleiomyomatosis. Horm Cancer.

[70] Sun Y (2014). Estradiol promotes pentose phosphate pathway addiction and cell survival via reactivation of Akt in mTORC1 hyperactive cells. Cell Death Dis.

[71] Finlay GA (2004). Estrogen-induced smooth muscle cell growth is regulated by tuberin and associated with altered activation of platelet-derived growth factor receptor-beta and ERK-1/2. J Biol Chem.

[72] Guenther GG (2014). Loss of TSC2 confers resistance to ceramide and nutrient deprivation. Oncogene.

[73] Chen J (2009). ToppGene suite for gene list enrichment analysis and candidate gene prioritization. Nucleic Acids Res.

[74] Howe SR (1995). Rodent model of reproductive tract leiomyomata. Establishment and characterization of tumor-derived cell lines. Am J Pathol.

[75] Howe SR (1995). Estrogen stimulation and tamoxifen inhibition of leiomyoma cell growth in vitro and in vivo. Endocrinology.

[76] Hong F (2008). mTOR-raptor binds and activates SGK1 to regulate p27 phosphorylation. Mol Cell.

[77] Hammad SM (2010). Blood sphingolipidomics in healthy humans: impact of sample collection methodology. J Lipid Res.

[78] https://combosyn.com/uat/pdf/CompuSyn_users_guide.pdf.

[79] Onda H (1999). Tsc2(+/-) mice develop tumors in multiple sites that express gelsolin and are influenced by genetic background. J Clin Invest.

[80] Woodrum C (2010). Comparison of three rapamycin dosing schedules in A/J Tsc2+/- mice and improved survival with angiogenesis inhibitor or asparaginase treatment in mice with subcutaneous tuberous sclerosis related tumors. J Transl Med.

[81] Kalogerou M (2012). T2 weighted MRI for assessing renal lesions in transgenic mouse models of tuberous sclerosis. Eur J Radiol.

