# Supplementary material for: Upregulation of acid ceramidase contributes to tumor progression in tuberous sclerosis complex
Source: JCI Insight. 2023 May 8;8(9):e166850. doi: 10.1172/jci.insight.166850 (PMC10243802; doi:10.1172/jci.insight.166850)

Supplemental Information

Upregulation of acid ceramidase contributes to tumor progression in Tuberous Sclerosis Complex

Aristotelis Astrinidis, Chenggang Li, Erik Y. Zhang, Xueheng Zhao, Shuyang Zhao, Minzhe Guo, Tasnim Olatoke, Ushodaya Mattam, Rong Huang, Alan G. Zhang, Lori Pitstick, Elizabeth J. Kopras, Nishant Gupta, Roman Jandarov, Eric P. Smith, Elizabeth Fugate, Diana Lindquist, Maciej M. Markiewski, Magdalena Karbowniczek, Kathryn A. Wikenheiser-Brokamp, Kenneth D. R. Setchell, Francis X. McCormack, Yan Xu, and Jane J. Yu

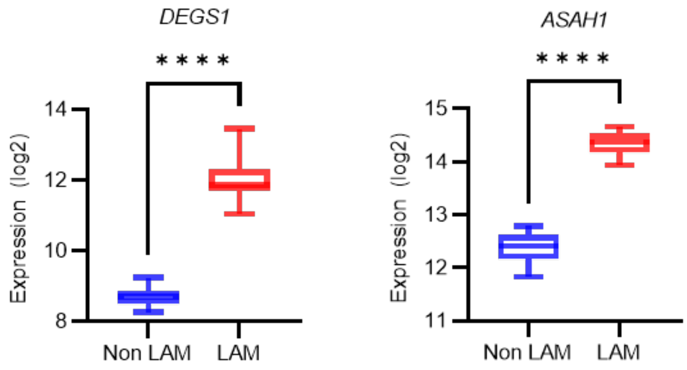

Supplemental Figure S1. Expression of *DEGS1* and *ASAH1* is evident in pulmonary LAM lesions. Box plots with 95% confidence intervals show *DEGS1* and *ASAH1* expression in female non-LAM lungs (n=15 subjects) and laser capture microdissected LAM lesion cells (n=14 subjects). **** P < 0.0001, Mann-Whitney test. (Corresponds to Fig. 2C).

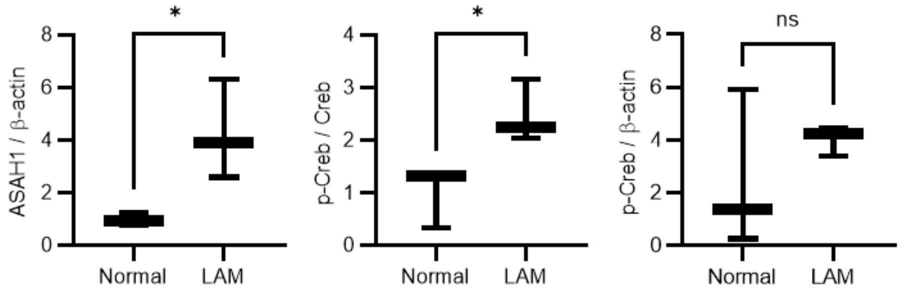

Supplemental Figure S2. Expression of ASAH1 and transcription factor CREB in LAM lungs.

Box plots with 95% confidence intervals show protein levels of ASAH1 and phospho-CREB in LAM (n=3) and control lungs (n=3) quantified by densitometry of ASAH1 and phospho-CREB. * $P < 0.05$, unpaired t test. (Corresponds to Fig. 2F).

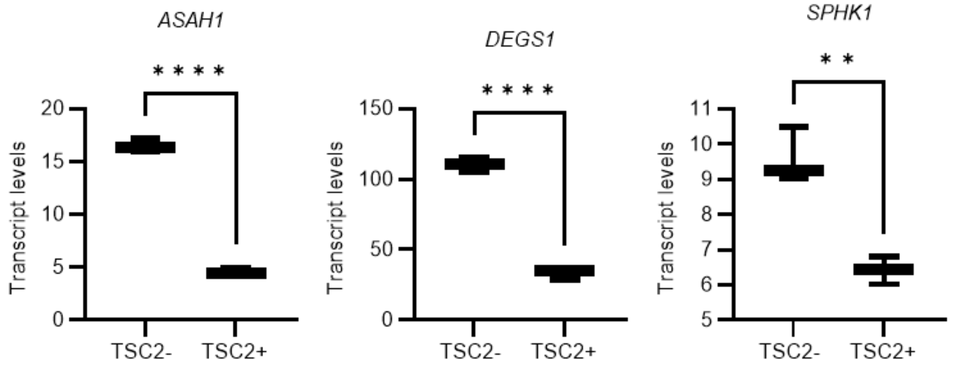

Supplemental Figure S3. Expression of sphingolipid biosynthesis pathway genes in TSC2-null cells. Box plots with 95% confidence intervals show mRNA levels of ASAH1, DEGS1, and SPHK1 in TSC2-null (TSC2-) 621-101 cells (n=3) and TSC2-addback (TSC2+) 621-103 cells (n=3). ** P < 0.01, **** P < 0.0001, unpaired t test. (Corresponds to Fig. 3B).

Supplemental Table S1. Statistical analyses and P values in *in vitro* and *in vivo* experiments.

Pairwise comparisons						
**Figure panel**	**Plot**	**Comparison groups (A vs. B)**	**Unpaired t test P value**	**Mann-Whitney test P value**	**n (A)**	**n (B)**
2C	ASAH1	Normal lung vs. LAM	**<0.0001**	**<0.0001**	15	14
	DEGS1	Normal lung vs. LAM	**<0.0001**	**<0.0001**	15	14
2F	ASAH1 / β-actin	Normal vs. LAM	**0.0411**	0.1	3	3
	p-CREB / CREB	Normal vs. LAM	**0.0377**	0.1	3	3
	p-CREB / β-actin	Normal vs. LAM	0.4387	0.7	3	3
3B	ASAH1	TSC2- vs. TSC2+	**<0.0001**	0.1	3	3
	DEGS1	TSC2- vs. TSC2+	**<0.0001**	0.1	3	3
	SPHK1	TSC2- vs. TSC2+	**0.0034**	0.1	3	3
3D	ASAH1 / β-actin	621-101 vs 621-103, 0% FBS	**0.0051**	0.1	3	3
		621-101 vs 621-103, 10% FBS	**<0.0001**	0.1	3	3
	DEGS1 / β-actin	621-101 vs 621-103, 0% FBS	**0.0063**	0.1	3	3
		621-101 vs 621-103, 10% FBS	**0.0008**	0.1	3	3
4A	Ceramide	TSC2- control vs. TSC2- rapa	0.2374 (0.4748)	0.1797 (0.3594)	6	6
		TSC2- control vs. TSC2+ control	**<0.0001** (**<0.0002**)	**0.0022** (**0.0044**)	6	6
4B	Sphingosine	TSC2- control vs. TSC2- rapa	0.4609 (0.9218)	0.4848 (0.9696)	6	6
		TSC2- control vs. TSC2+ control	**<0.0001** (**<0.0002**)	**0.0022**	6	6
4C	ASAH1 transcript	TSC2- control vs. TSC2- rapa	0.7447 (1.0)	>0.9999 (1.0)	6	6
		TSC2- control vs. TSC2+ control	**<0.0001** (**<0.0002**)	**0.0022** (**0.0044**)	6	6
4F	cAMP levels	TSC2- vs TSC2+	**0.0098**	0.1	3	3
4J	ASAH1 / β-actin	Vehicle vs. AZD6244	**<0.0001** (**<0.0003**)	0.1 (0.3)	3	3
		Vehicle vs. L798106	**<0.0001** (**<0.0003**)	0.1 (0.3)	3	3
		Vehicle vs. PKI	**<0.0001** (**<0.0003**)	0.1 (0.3)	3	3
4K	ASAH1 transcript	Vehicle vs. E2 (10 nM)	**0.0002**	0.1	3	3
4N	ERa / β-actin	Vehicle vs. E2 24h	**0.0014**	0.1	3	3
	ASAH1 / β-actin	Vehicle vs. E2 24h	**0.0019**	0.1	3	3
5A	17a 50 nM	TSC2- vs. TSC2+	**<0.0001**	ND	4	4
	17a 100 nM	TSC2- vs. TSC2+	**<0.0001**	ND	4	4
	17a 200 nM	TSC2- vs. TSC2+	**<0.0001**	ND	4	4
5B	Carmofur 10 μM	TSC2- vs. TSC2+	**0.0038**	**0.0286**	4	4
	Carmofur 20 μM	TSC2- vs. TSC2+	**0.0033**	**0.0286**	4	4
	Carmofur 50 μM	TSC2- vs. TSC2+	**<0.0001**	**0.0286**	4	4
	Carmofur 100 μM	TSC2- vs. TSC2+	**0.0003**	**0.0286**	4	4

5C	Gene expression	siCtrl vs. siASAH1	**0.0019**	**0.0286**	4	4
5E	Cell viability 48 h post	siCtrl vs. siASAH1	**<0.0001**	**<0.0001**	12	12
5G	Apoptotic Cells	siASAH1- vs. siASAH1+	**<0.0001** (**<0.0002**)	0.1 (0.2)	3	3
		siASAH1- vs. siASAH1+ Ceramide	**<0.0001** (**<0.0002**)	0.1 (0.2)	3	3
5I	Cell viability	pLKO.1 vs. shASAH1 #1	**<0.0001** (**<0.0002**)	**<0.0001** (**<0.0002**)	15	16
		pLKO.1 vs. shASAH1 #2	**<0.0001** (**<0.0002**)	**<0.0001** (**<0.0002**)	15	16
5K	Apoptosis	pLKO.1 vs. shASAH1 #1	**0.0001 (0.0002)**	0.1 (0.2)	3	3
		pLKO.1 vs. shASAH1 #2	**<0.0001** (**<0.0002**)	0.1 (0.2)	3	3
5L	Cell death	pLKO.1 vs. shASAH1 #1	**<0.0001** (**<0.0002**)	**<0.0001** (**<0.0002**)	15	16
		pLKO.1 vs. shASAH1 #2	**<0.0001** (**<0.0002**)	**<0.0001** (**<0.0002**)	15	16
6B	Bioluminescence imaging	Vehicle vs. 17a, week 4	**0.0441**	**0.0260**	6	6
6F	Bioluminescence imaging	pLKO.1 vs. shASAH1, week 22	**0.0020**	**0.0357**	3	5
		pLKO.1 vs. shASAH1, week 23	**0.0258**	**0.0357**	3	5
6H	Bioluminescence imaging	Vehicle vs. 17a, 6 hours	**0.0368**	**0.0286**	3	3
		Vehicle vs. 17a, 24 hours	**0.0341**	**0.0286**	3	3
7B	Macroscopic cysts	Control vs. rapa	**0.0345** (0.1035)	**0.0286** (0.0858)	4	4
		Control vs. 17a+rapa	**0.0071** (**0.0213**)	**0.0286** (0.0858)	4	4
		rapa vs. 17a+rapa	**0.0100** (**0.0300**)	**0.0286** (0.0858)	4	4
**Ordinary one-way ANOVA with Tukey's multiple comparisons test**						
**Figure panel**	**Plot**	**Comparison groups (A vs. B)**	**Adj. P value**		**n (A)**	**n (B)**
7F	Tumor burden 4 weeks	17a vs. rapa	**0.0011**		5	2
		17a vs. 17a+rapa	**0.0001**		5	5
		rapa vs. 17a+rapa	0.9992		2	5
	Tumor burden 8 weeks	17a vs. rapa	0.1562		4	2
		17a vs. 17a+rapa	**0.0013**		4	5
		rapa vs. 17a+rapa	0.1087		2	5
7H	Percent PCNA	Control vs. Rapamycin	0.0967		5	5
		Control vs. 17a	**0.0035**		5	5
		Control vs. Rapamycin+17a	**<0.0001**		5	5
		Rapamycin vs. 17a	**<0.0001**		5	5
		Rapamycin vs. Rapamycin+17a	**<0.0001**		5	5
		17a vs. Rapamycin+17a	**0.0395**		5	5
**Two-way ANOVA with Tukey's multiple comparisons test**						
**Figure panel**	**Plot**	**Comparison groups (A vs. B)**	**Adj. P value**		**n (A)**	**n (B)**
6J	Bioluminescence imaging 6 h	pLKO.1 vs. shASAH1 #1	**0.0013**		4	4

		pLKO.1 vs. shASAH1 #2	**<0.0001**		4	5
		shASAH1 #1 vs. shASA1 #2	0.3537		4	5
	Bioluminescence imaging 24 h	pLKO.1 vs. shASAH1 #1	**0.0414**		4	4
		pLKO.1 vs. shASAH1 #2	**0.0005**		4	5
		pLKO.1 vs. shASAH1 #2	0.2631		4	5
**Survival**						
**Figure panel**	**Plot**	**Comparison groups (A vs. B)**	**Log-rank (Mantel-Cox) test P value**			
6E	Tumor-free survival	pLKO.1 vs. shASAH1 #1	**0.0493**			
		pLKO.1 vs. shASAH1 #2	**0.0002**			
		shASAH1 #1 vs. shASAH1 #2	**0.0234**			

Numbers in parenthesis represent P values between comparison groups after Bonferroni multiple comparison adjustment. ND = not determined

Supplemental Table S2. Normality and Lognormality tests for all data groups presented in Fig. 3B.

	ASAH1		*DEGS1*		*SPHK1*	
**Test**	**TSC2-**	**TSC2+**	**TSC2-**	**TSC2+**	**TSC2-**	**TSC2+**
D'Agostino & Pearson	N too small	N too small	N too small	N too small	N too small	N too small
Anderson-Darling	N too small	N too small	N too small	N too small	N too small	N too small
**Shapiro-Wilk**	**PASS (P= 0.6805)**	**PASS (P= 0.0587)**	**PASS (P= 0.8759)**	**PASS (P= 0.2738)**	**PASS (P= 0.2720)**	**PASS (P= 0.9511)**
Kolmogorov-Smirnov	N too small	N too small	N too small	N too small	N too small	N too small

Supplemental Table S3. Normality and Lognormality tests for all data groups presented in Fig. 3D for ASAH1 / β-actin.

**Test**	**TSC2- 0% FBS**	**TSC2+ 0% FBS**	**TSC2- 10% FBS**	**TSC2+ 10% FBS**
D'Agostino & Pearson	N too small	N too small	N too small	N too small
Anderson-Darling	N too small	N too small	N too small	N too small
**Shapiro-Wilk**	**PASS (P= 0.2968)**	**PASS (P= 0.3554)**	**PASS (P= 0.4014)**	**PASS (P= 0.1720)**
Kolmogorov-Smirnov	N too small	N too small	N too small	N too small

Supplemental Table S4. Normality and Lognormality tests for all data groups presented in Fig. 3D for DEGS1 / β-actin.

**Test**	**TSC2- 0% FBS**	**TSC2+ 0% FBS**	**TSC2- 10% FBS**	**TSC2+ 10% FBS**
D'Agostino & Pearson	N too small	N too small	N too small	N too small
Anderson-Darling	N too small	N too small	N too small	N too small
**Shapiro-Wilk**	**PASS (P= 0.3079)**	**PASS (P= 0.3231)**	**PASS (P= 0.7637)**	**PASS (P= 0.6702)**
Kolmogorov-Smirnov	N too small	N too small	N too small	N too small

Figure 2

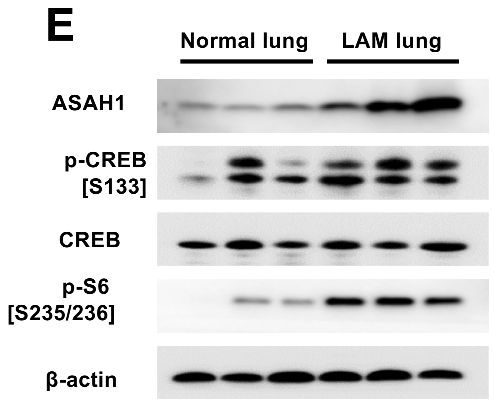

Uncropped gel images

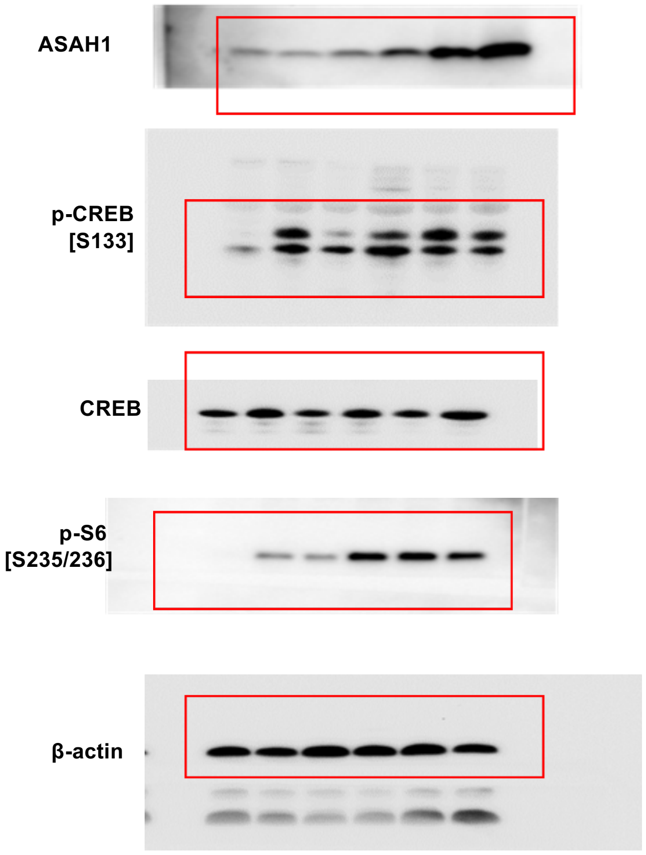

Figure 3 C

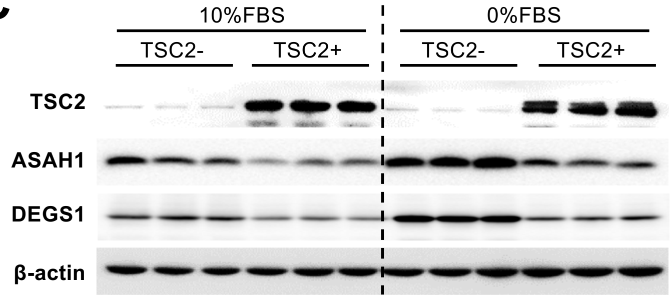

Human renal angiomyolipoma-derived cells

Uncropped gel images

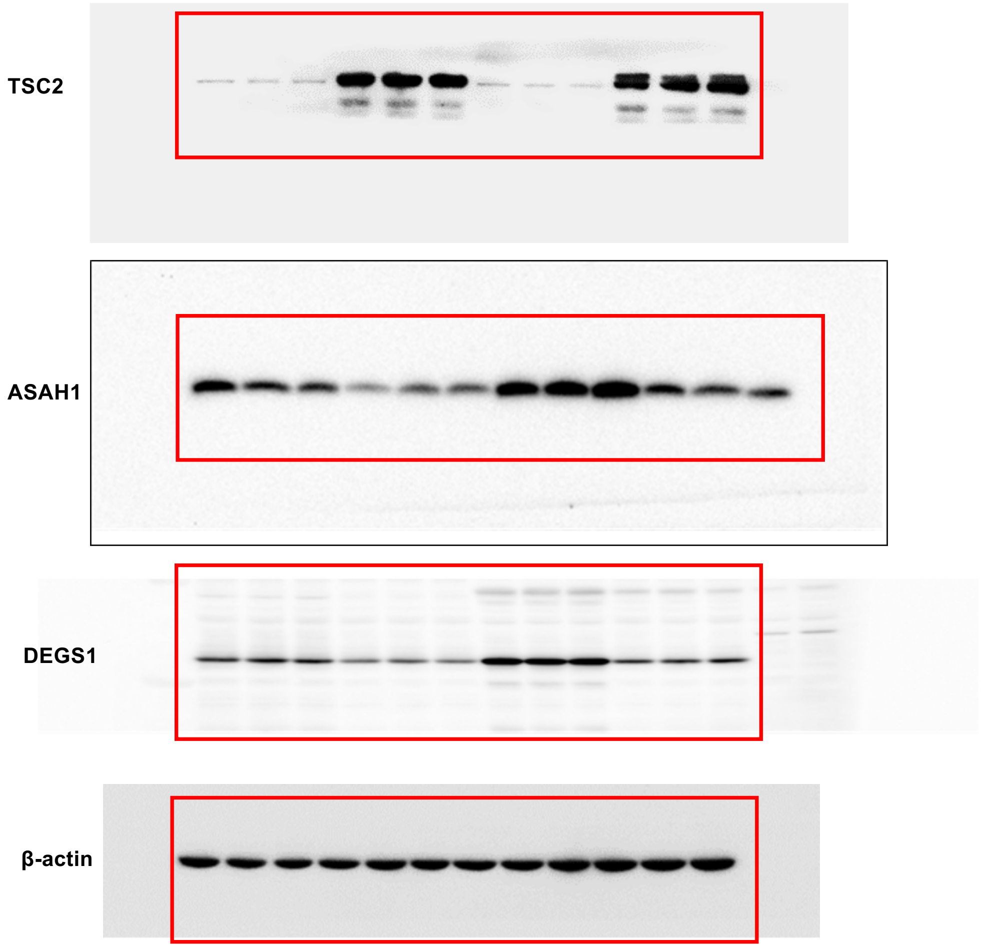

Revised Figure 4

D

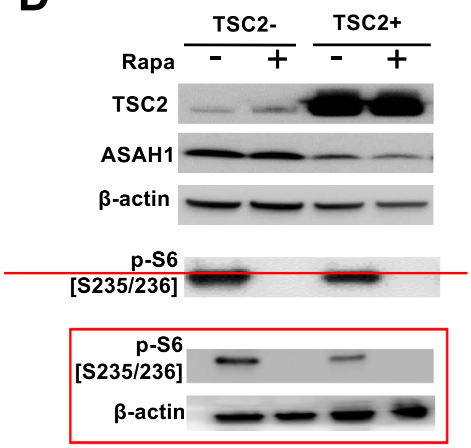

Uncropped gel images

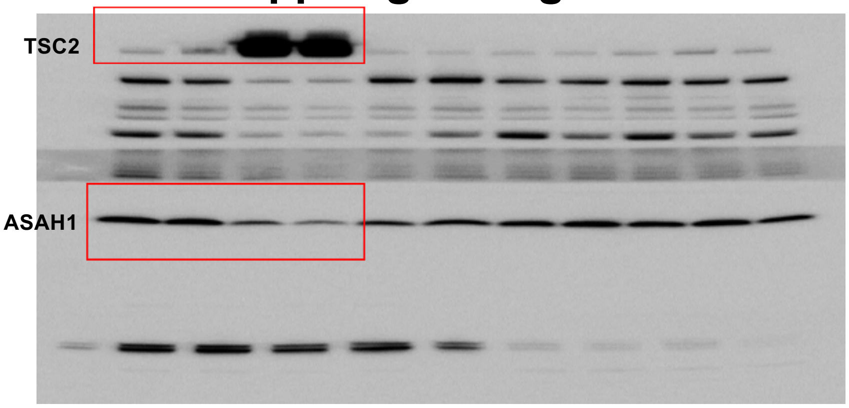

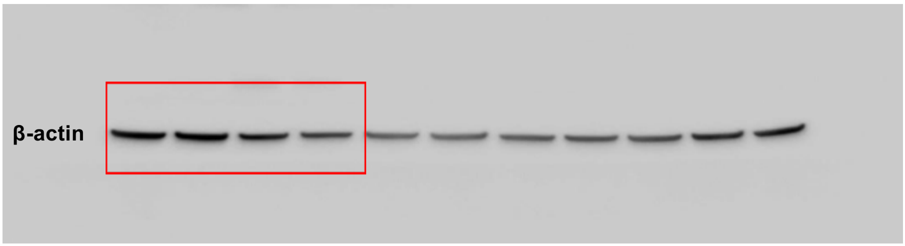

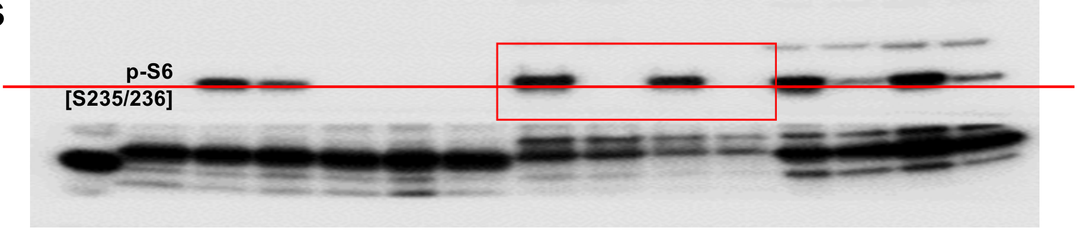

Revision
Uncropped gel images

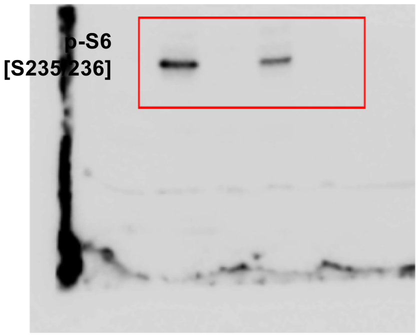

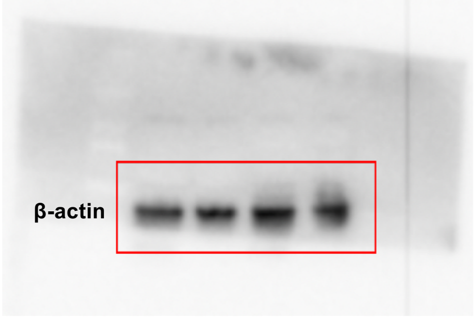

Figure 4

E

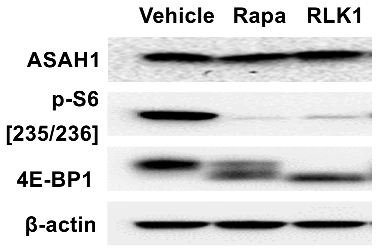

Uncropped gel images

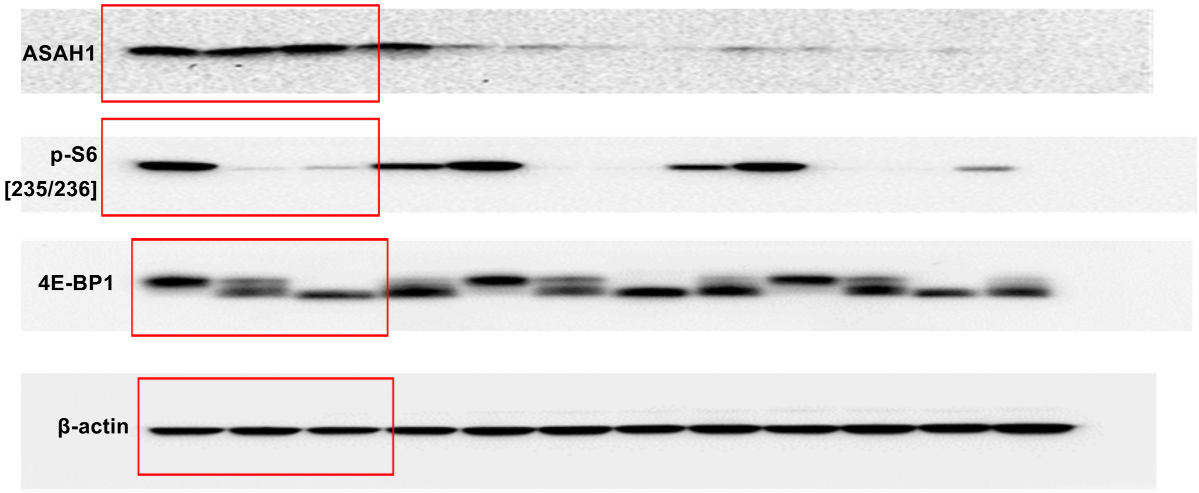

Figure 4

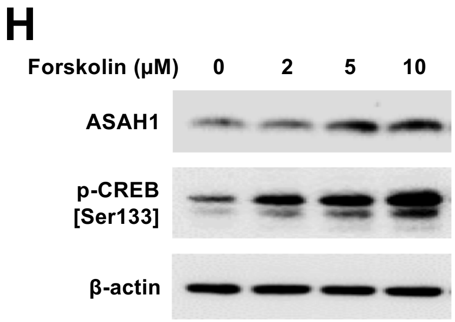

Uncropped gel images

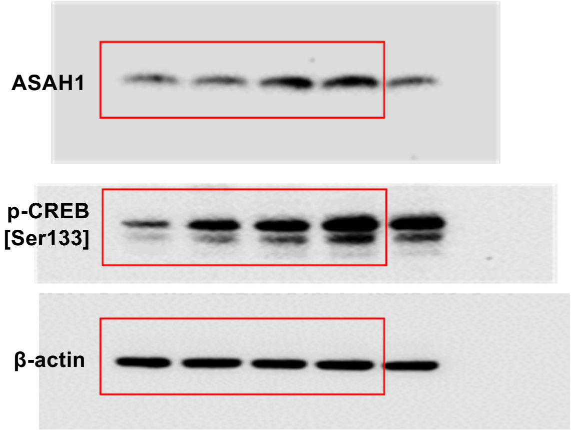

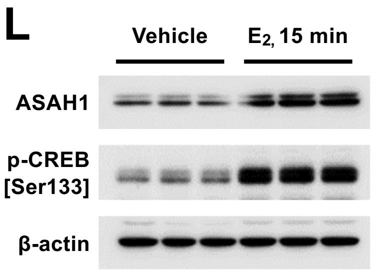

Uncropped gel images

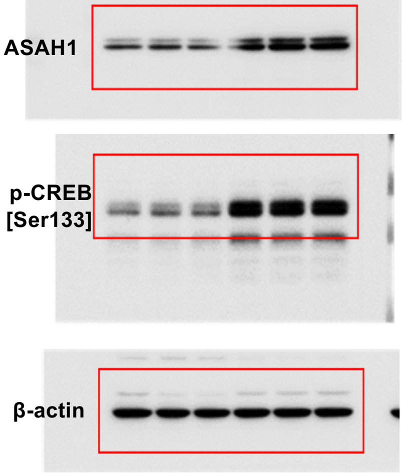

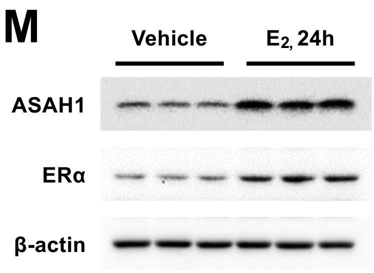

Uncropped gel images

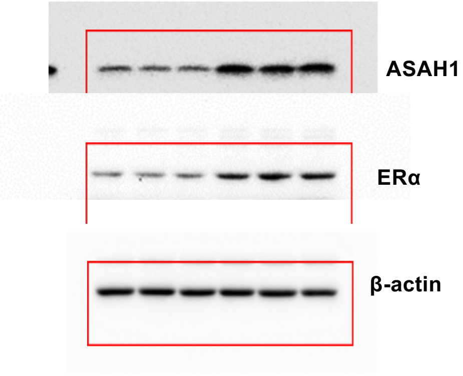

Figure 4

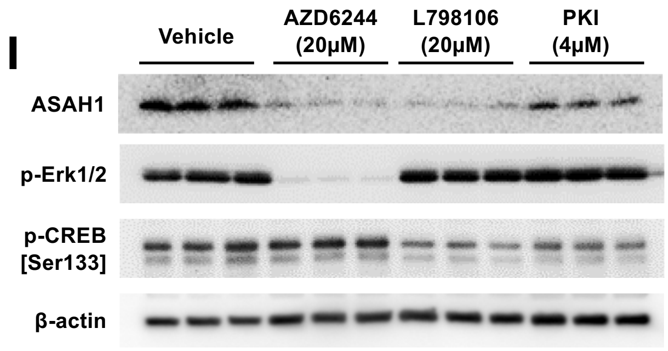

Uncropped gel images

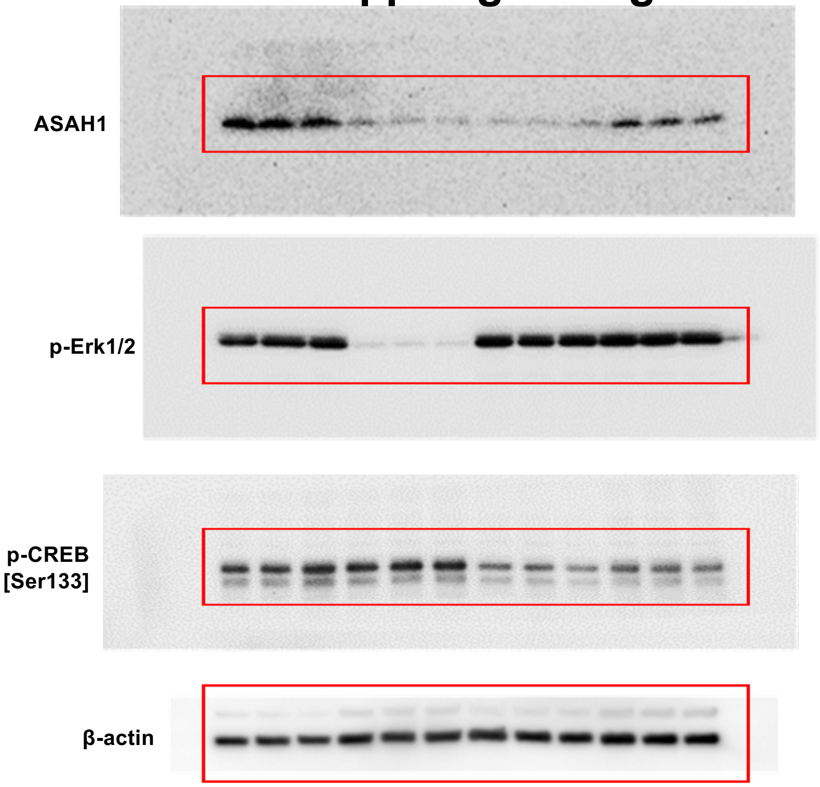

New Figure 5D

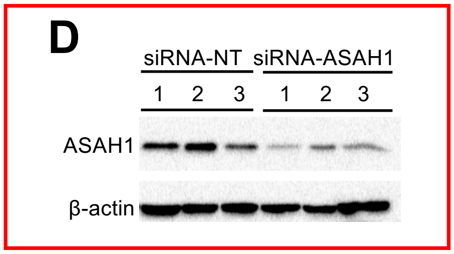

Uncropped gel images

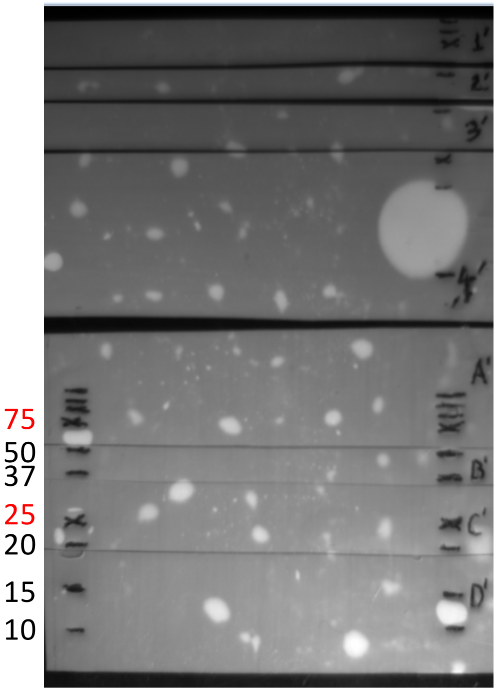

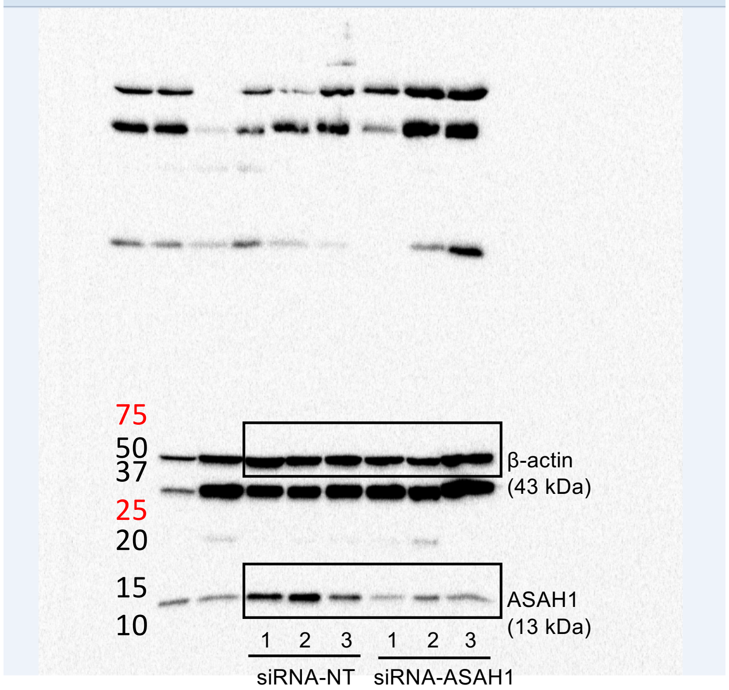

Figure 5

H

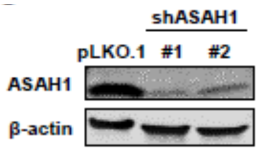

621-101 cells

Uncropped gel images

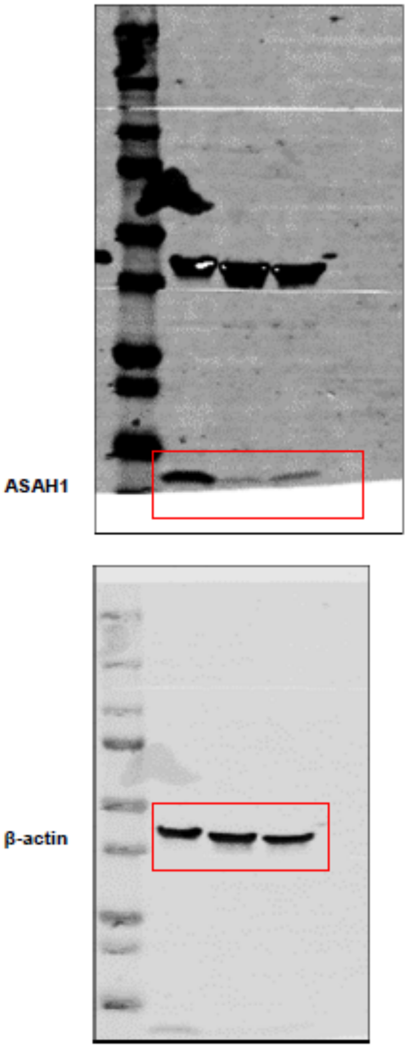

Figure 6

C

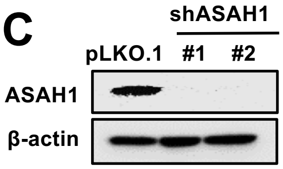

LAM patient–derived 621-101 cells

Uncropped gel images

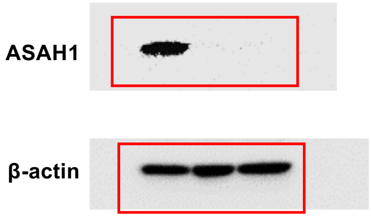

Supplement: Supplemental data [file jciinsight-8-166850-s037.pdf]
